# Supplementary material for: Modeling Motor Neuron Resilience in ALS Using Stem Cells
Source: Stem Cell Reports. 2019 May 9;12(6):1329–41. doi: 10.1016/j.stemcr.2019.04.009 (PMC6565614; doi:10.1016/j.stemcr.2019.04.009)
Supplement: Document S1. Supplemental Experimental Procedures and Figures S1–S4 [file mmc1.pdf]

Stem Cell Reports, Volume *12*

Supplemental Information

Modeling Motor Neuron Resilience in ALS Using Stem Cells

Ilary Allodi, Jik Nijssen, Julio Aguila Benitez, Christoph Schweingruber, Andrea Fuchs, Gillian Bonvicini, Ming Cao, Ole Kiehn, and Eva Hedlund

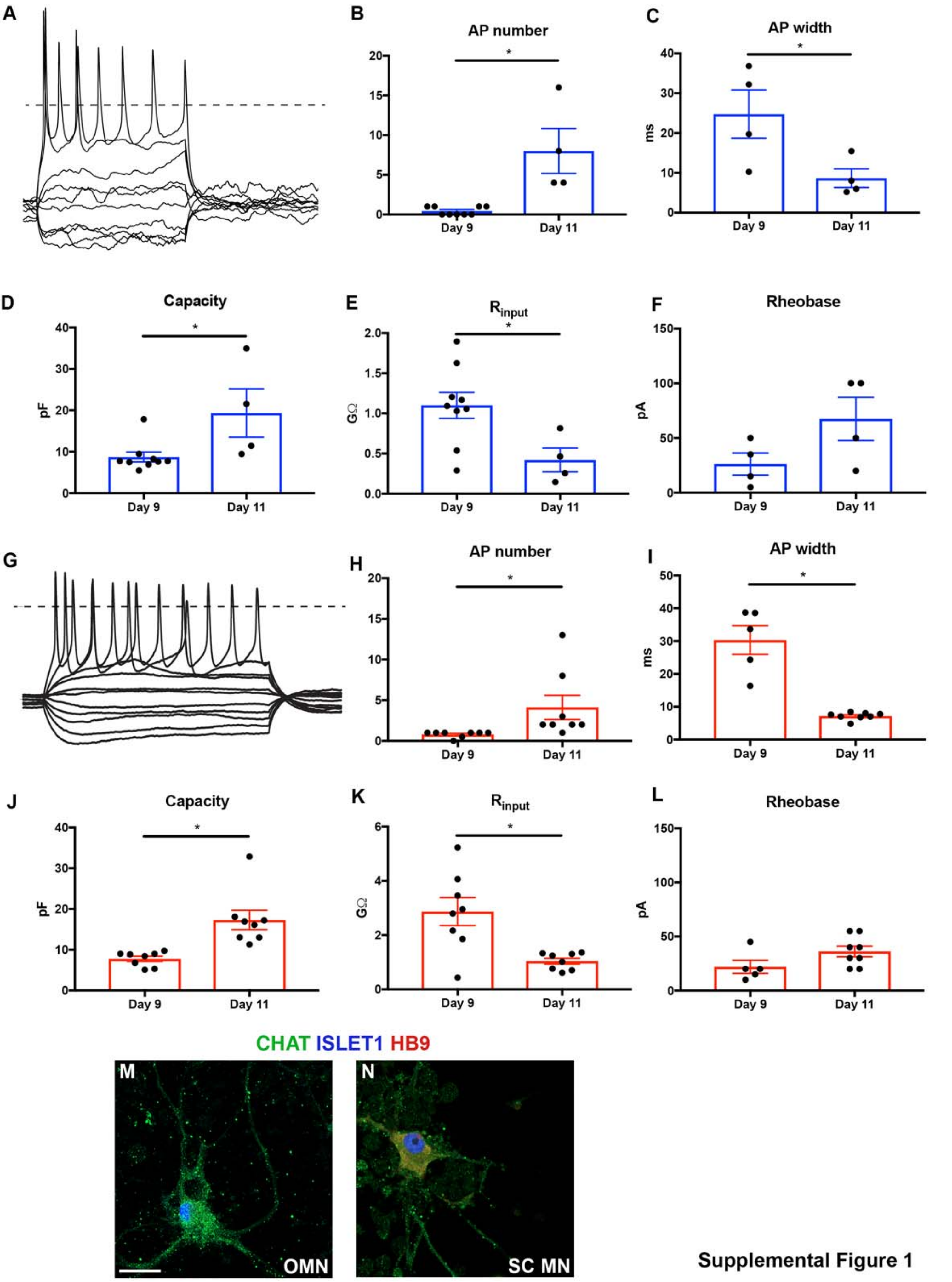

Supplemental Figure 1

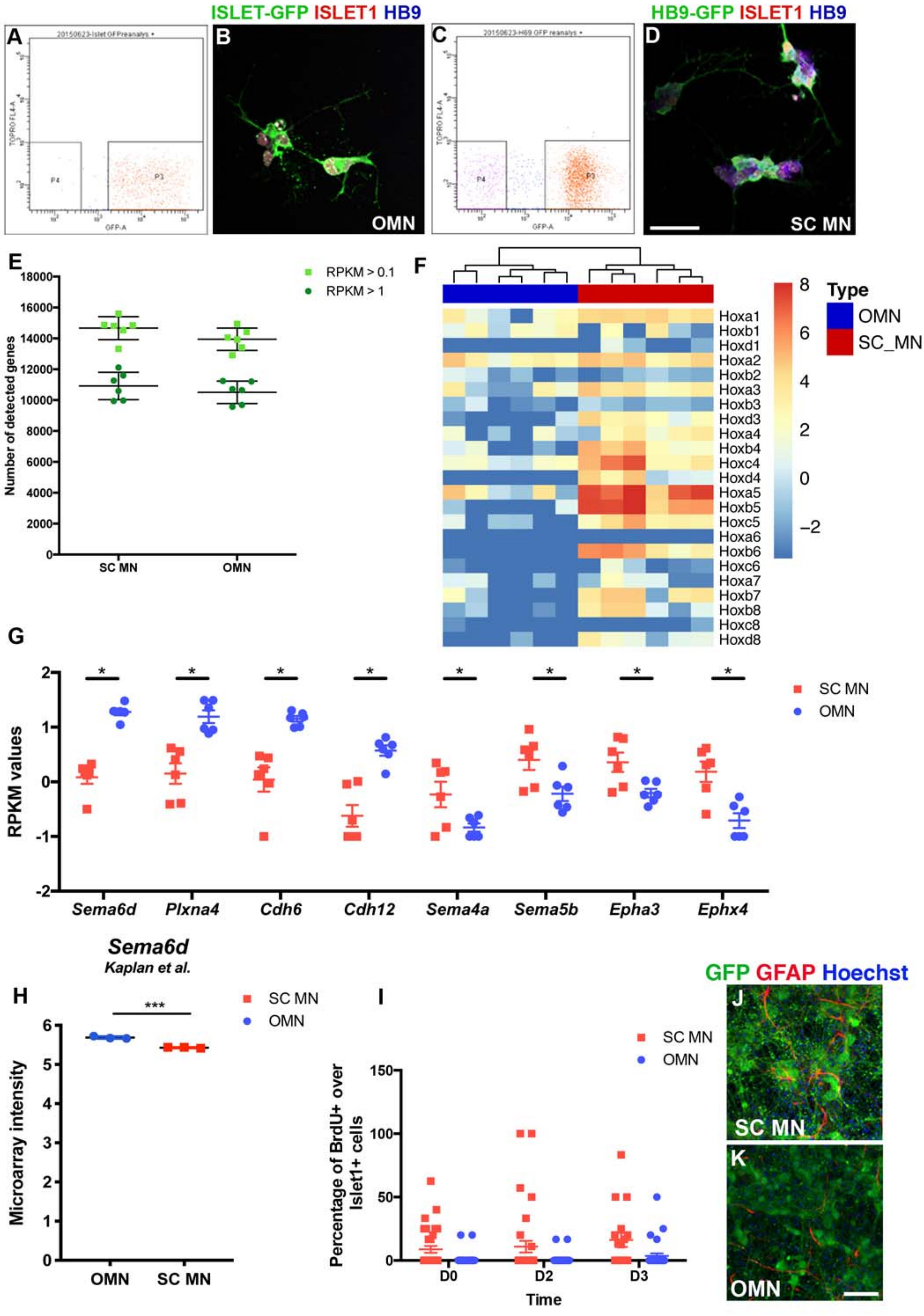

Supplemental Figure 2

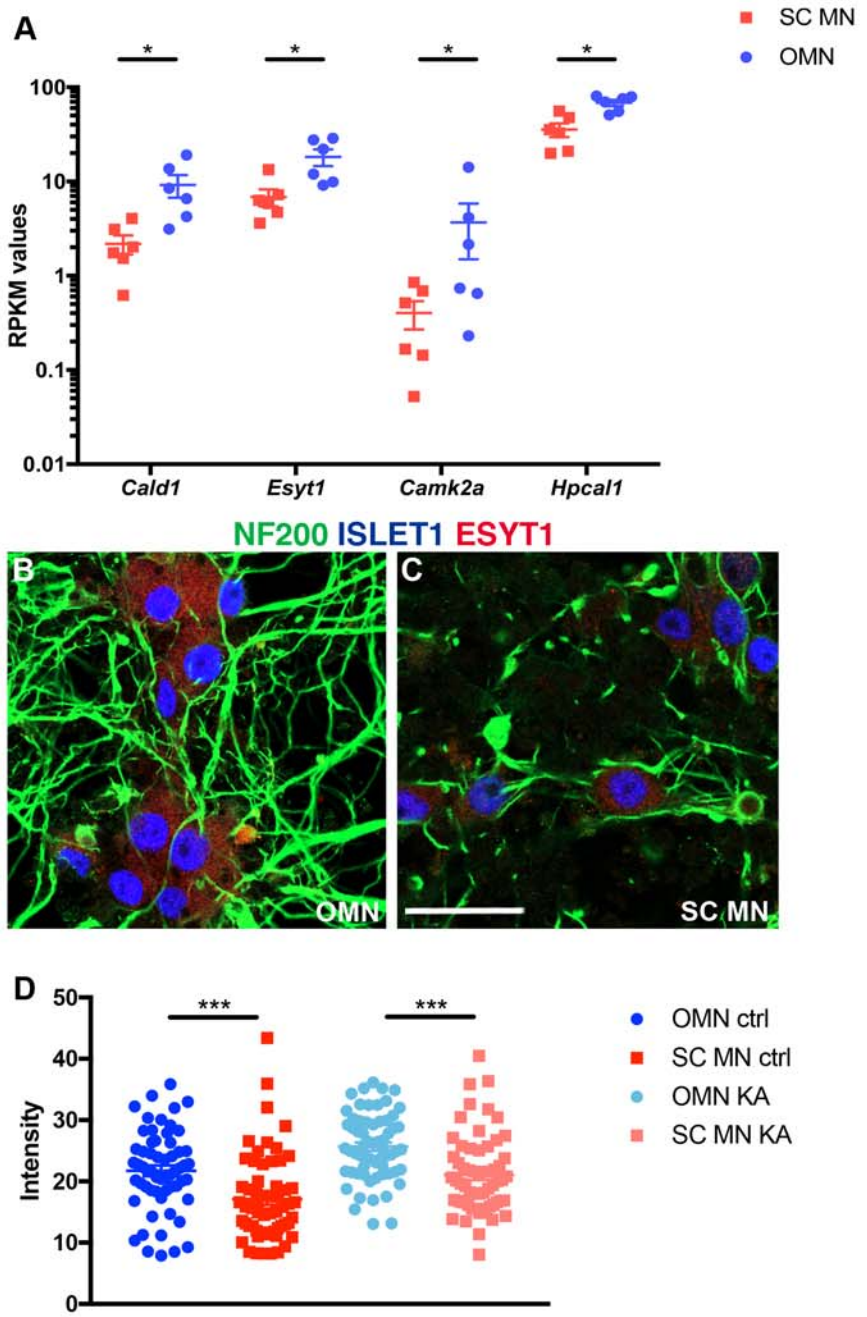

Supplemental Figure 3

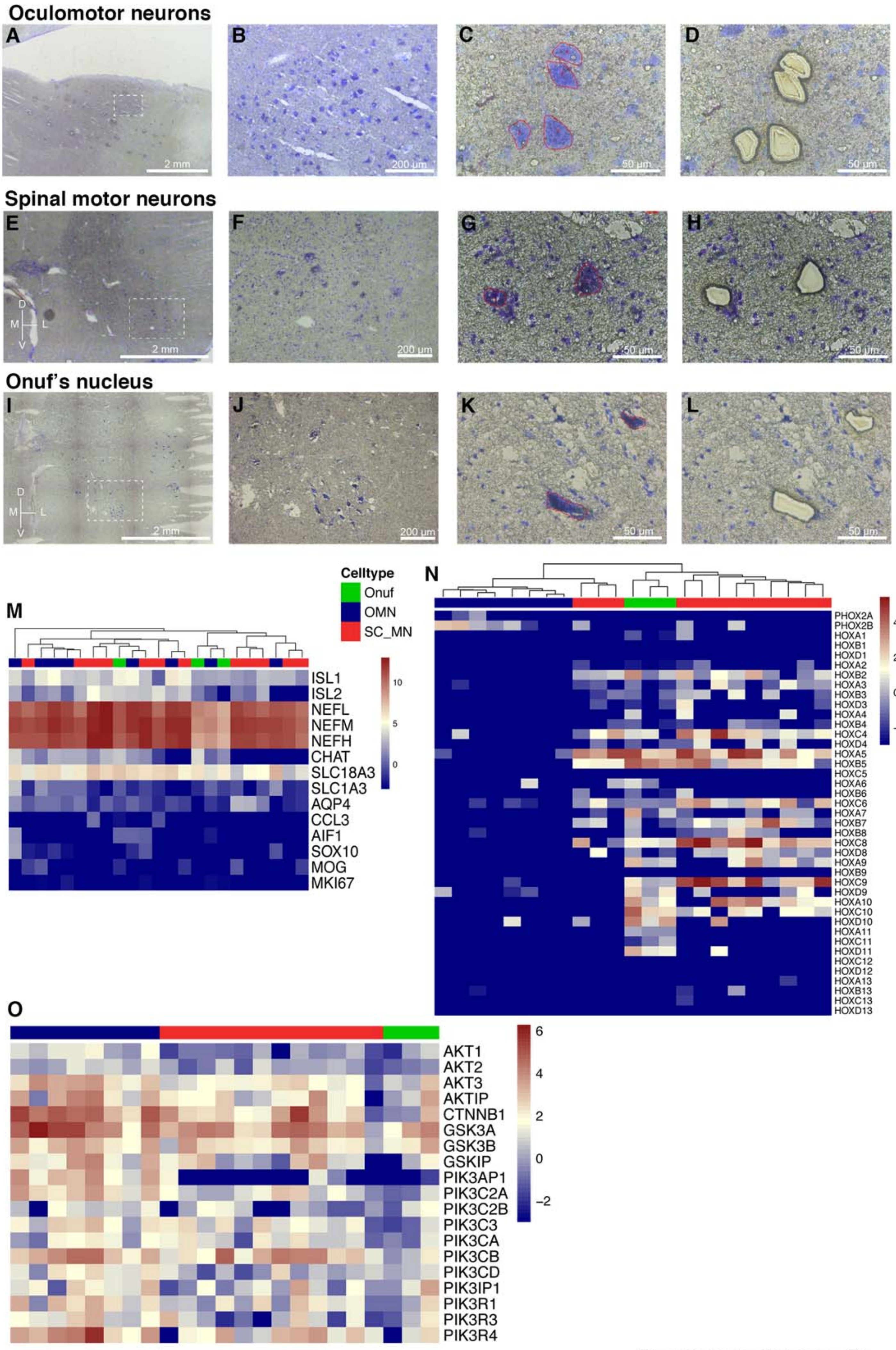

Supplemental Figure S4

Supplemental Figure S1. Oculomotor neurons and spinal motor neurons develop electrical properties and mature in culture. Cells measured in whole cell configuration in current clamp mode, example traces at day 11 for OMN and SC MN cultures (A; G). Dotted lines indicate 0 mV (A; G). Voltage response to step current injections (-40 pA increasing in 10 pA step) at day 11 in OMNs (A). Increased number of action potentials at day 11 compared to day 9 (B, t test, P= 0.0015, mean±SEM) and reduced width of action potentials (C, t test, P=0.0475, mean±SEM). OMNs at day 11 (n=4) show increased cell capacity (D, t test, P=0.0252, mean±SEM), decreased input resistance (E, t test, P= 0.0270, mean±SEM) and increased rheobase (F, t test, mean±SEM) as compared to Day 9 OMNs (n=9). Voltage response to step current injections (-25 pA increasing in 5 pA steps) at day 11 in SC MN (G). Number of action potentials (H, t test, P=0.0428, mean±SEM) and action potential width (I, t test, P<0.0001, mean±SEM) at day 9 and day 11 in SC MNs. Increased cell capacity (J, t test, P=0.0017, mean±SEM), decreased input resistance (K, t test, P=0.0037, mean±SEM) and rheobase (L, t test, mean±SEM) at day 11 as compared to day 9. CHAT staining in OMNs (M) and SC MNs (N) at day 14 in vitro. CHAT in green, HB9 in red, ISLET1 in blue. Scale bar = 60 μm.

Supplemental Figure S2. Generation of oculomotor neurons and spinal motor neurons from stem cells and their characterization. (A) FACS reanalysis plots of *Hb9*-GFP and (C) *Islet1*-GFP/*NesEPhox2*a cells sorted based on GFP expression demonstrated an enrichment of motor neurons (MNs) (*Hb9*-GFP 85%, *Islet*-GFP/*NesEPhox2*a 96% in P3 plots). (B) FAC sorted spinal motor neurons (SC MNs) express ISLET1 and HB9, while oculomotor neurons (OMNs) express ISLET1, while lacking HB9 (D) scale bar = 60 μm. (E) Graph showing mean number of detected genes (and RPKM > 1) (mean ± SEM, t test for RPKM > 0.1 P=0.1196, t test for RPKM > 1 P=0.4047, n=6) after mRNA-seq analysis performed on EB dissociation day following FACS of *Hb9*GFP+ (SC MNs) and *Islet1*GFP/*NesEPhox2*a+ (OMNs) cells. Both conditions show similar amounts of expressed genes. (F) Heatmap showing the differential Hox gene expression between OMNs and SC MNs, confirming their positional identity. (G) DESEQ analysis performed on transcripts obtained from generated oculomotor neurons (OMNs) and spinal motor neurons (SC MNs) reveal significant differences in genes controlling axon guidance, Log_{10}-transformed data for *Sema6d*, *Plxa4*, *Cdh6* and *Cdh12*, preferentially expressed in OMNs, and *Sema4a*, *Sema5b*, *Epha3* and *Ephx4* preferentially expressed in SC MNs (adjusted *P < 0.05, n=6). (H) Preferential expression of *Sema6d* in oculomotor neurons in the published Kaplan et al data set (t test, *P* <0,001). (I) Graph indicated percentage of BrdU+ cells over ISLET1+ cells at D7 assay. BrdU pulses were performed at D0, D2 and D3 survival assay quantifications were performed at D7. (mean ± SEM, 2way ANOVA and Tukey's multiple comparison test, F(2, 169)=0.3278, P=0.7209, n=3). 10 random areas were quantified per each time point per experiment (in duplicates), with at least 100 cells quantified per condition.

Supplemental Figure S3. Preferential expression of Ca^{2+}-regulating genes in oculomotor neurons. (A) After DESEQ analysis of generated oculomotor neuron (OMN) and spinal motor neuron (SC MNs) mRNA expression, several transcripts of Ca^{2+} binding proteins could be found differentially expressed. Graph shows four of them (*Cald1*, *Esyt1*, *Camk2a*, *Hpcal1*) with preferential expression in OMNs suggesting increased capability of intracellular Ca^{2+} buffering during excitotoxicity (adjusted $*P<0.05$, log_{10}-transformed RPKM values, n=6). (B-C) ESYT1 immunohistochemistry performed on OMNs and SC MNs at D7 toxicity assay revealed preferential expression in OMNs at protein level. Scale bar = 60 µm. (D) Semi-quantification of ESYT1 staining in control and KA20 conditions (mean ± SEM, 2way ANOVA and Tukey's multiple comparison test, $F(1, 239)=31,84$, *** $P < 0.0001$, n=3). Experiments were performed with technical replicates and with at least 100 motor neurons counted per condition.

Supplemental Figure S4. LCM-seq of human resilient and vulnerable motor neuron groups.
Laser capture microdissection (LCM) was used to isolated oculomotor neurons (A-D), spinal (cervical and lumbar) motor neurons (E-H) and Onuf's nucleus motor neurons (I-L) from human *post mortem* tissues. RNA sequencing of isolated neurons showed that (M) motor neurons also expressed *ISLET1/2* and *CHAT*, while being almost devoid of contaminating glial markers. (N) Analysis of the *PHOX2A/B* and *HOX* gene expression clustered oculomotor neurons away from all spinal motor neuron groups. (O) Analysis of the *PI3K-AKT* signaling pathway.

Supplemental Experimental Methods

Electrophysiology

*Islet1*GFP/*NesEPhox2a* and *Hb9*GFP cell lines were used to visualize oculomotor and spinal cord motor neurons, respectively. EBs were dissociated and cells were plated onto glass coverslips, coated with Poly-L-ornitine (0.001%, Gibco) and fibronectin (1ug/ml, Gibco), at a density of 200,000 cells per well (24-well plate). The coverslips were placed in a recording chamber and where superfused with with oxygenated recording buffer (95% O_2/5% CO_2, 22-24 °C), composed of 111 mM NaCl, 3 mM KCl, 26 mM $NaHCO_3$, 1.1 mM KH_2PO_4, 2.5 mM $CaCl_2$, 1.25 mM $MgCl_2$, and 11 mM D-glucose. Patch pipettes (5-6 MΩ) were pulled from borosilicate glass (GC150F-10, Harvard Apparatus) and filled with (in mM): 128 KGlu, 4 NaCl, 10 HEPES, 0.0001 $CaCl_2$, 1 glucose, 5 ATP, 0.3 GTP, pH 7.4. Neurons were visualized by video microscopy (Axioskop 2 FS plus, Zeiss) using transversal illumination for increasing contrast and motor neurons were identified under fluorescence using a GFP filter. Whole-cell recordings were performed using a MultiClamp 700A amplifier (Molecular Devices) and acquired using pClamp software (Clampex v.9.2, Molecular Devices). Data were sampled at 20 kHz. Input resistance was measured from the I-V curves; the time constant was fitted with a single exponential curve and the capacity was calculated from the cell time constant and input resistance. The width of the action potential was measured at the half peak of the action potential. Rheobase was defined as the minimal current injection needed to elicit an action potential.

Extended laser capture microdissection and RNA sequencing of human motor neurons

Midbrain and spinal cord tissues were sectioned at 10 µm thickness, collected onto MembraneSlide 1.0 PEN microscope slides (Zeiss) and subjected to a quick histological Nissl stain based on the Arcturus Histogene Staining Kit protocol. Slides were then placed under a Leica DM6000R/CTR6500 microscope and captured using the Leica LMD7000 system. Motor neurons were identified by their soma size and their distinct location in axial tissue sections. The cutting outlines were drawn closely around individual cells order to diminish any contamination by surrounding cell. Pools of 20-150 cells were collected and directly lysed in 5µl of 0.2% triton X-100 (Sigma-Aldrich) with RNAse inhibitor (1.5U, Takara). After lysis samples were immediately frozen on dry ice until further processing. Library preparation for sequencing on Illumina HiSeq2000 or HiSeq2500 sequencers was carried out following a modified Smart-seq2 protocol (Nichterwitz et al., 2016).

Extended RNA-seq data processing

For the human LCM data, independent LCM samples obtained from tissue originating from the same individual (biological replicates) were pooled at the counts level. This ensured that each sample used in analysis was derived from one distinct individual. Ultimately, all 12 spinal cord samples were derived

from distinct individuals. Six oculomotor neuron samples originated from distinct individuals, while four more were summed from a total of 12 LCM-seq samples, resulting in a total n=10 samples (from ten individuals) for oculomotor neurons. Two samples were subsequently excluded due to their lack of motor neuron markers, as described above, resulting in a total of eight oculomotor samples. For Onuf's nucleus, three samples (from three individuals) were used in the analysis, derived from a total of seven LCM-seq samples.

Differential gene expression analysis was performed with the DESeq2 package in R on the raw read counts (version 1.16.1) (Love et al., 2014). Only genes with counts in at least 2 samples were considered for further analysis. No fold change cutoff was implemented and multiple testing correction was performed using the Benjamini & Hochberg correction with an FDR set to 10%. Adjusted p-values < 0.05 were considered significant. Pathway And Gene set OverDispersion Analysis (PAGODA) was run using the SCDE package in R (Fan et al., 2016). All heatmaps were generated in R with either no clustering or *Euclidean distance* where appropriate.

Immunocytochemistry and immunohistochemistry

Coverslips were fixed in 4% paraformaldehyde in 0.1M phosphate buffer (4% PFA) for 20 minutes, washed with PBS and then blocked with blocking solution composed of 10% normal donkey serum or normal goat serum (both from Thermo Fisher Scientific Inc.) and 0.1% Triton-X-100 in PBS for one hour at room temperature. Coverslips were incubated overnight with primary antibodies in blocking solution at 4˚C and then incubated with Alexa Fluorophore 488, 568 or 647 secondary antibodies (Invitrogen) one hour at room temperature. The following primary antibodies were used: NF200 (1:1000, #AB5539 Millipore), TUJ1 (1:1000, MRB-435P Covance), HB9 (1:10, #81.5C10 DSHB), ISLET1 (1:500, #ab20670 abcam; 1:100, #40.2D6 DSHB), PHOX2A (1:1000, gift of Prof JF Brunet), GFP (1:1000, #ab13970 abcam), phospho-AKT (Ser473) (1:50, #3787 Cell Signaling), activated BETA-CATENIN (1:1000, #05-665 Millipore), alpha-CATENIN (1:1000, #AB153721 AbCam), ESYT1 (1:100, #HPA016858), BrdU (1:10, #G3G4 DSHB). Hoechst-33342 (Invitrogen) was used as a counterstain. Finally, coverslips were mounted on glass slides using Mowiol 4-88 mounting media (Calbiochem). For immunocytochemistry semi-quantifications and BrdU quantifications at least 3 experiments per condition were counted in technical duplicates. Between 10 and 12 random areas were analyzed per coverslip by blind researcher. For validation of ESYT1 antibody, immunohistochemistry was performed on adult mouse tissue at midbrain and spinal cord levels. Briefly, mice were transcardially perfused with PBS and then with 4% PFA. Brains and spinal cords were dissected and post-fixed for 3 and 1 hour respectively in 4% PFA. Tissue was then cryoprotected with 30% sucrose and then cut at 30 μm thickness. Immunohistochemistry for ESYT1 was performed as indicated above.

Use of published datasets

Microarray data from Kaplan et al. (2014) was obtained through GEO accession number GSE52118.

References

Fan, J., Salathia, N., Liu, R., Kaeser, G.E., Yung, Y.C., Herman, J.L., Kaper, F., Fan, J.B., Zhang, K., Chun, J., *et al.* (2016). Characterizing transcriptional heterogeneity through pathway and gene set overdispersion analysis. Nat Methods *13*, 241-244.

Kaplan, A., Spiller, K.J., Towne, C., Kanning, K.C., Choe, G.T., Geber, A., Akay, T., Aebischer, P., and Henderson, C.E. (2014). Neuronal matrix metalloproteinase-9 is a determinant of selective neurodegeneration. Neuron *81*, 333-348.

Love, M.I., Huber, W., and Anders, S. (2014). Moderated estimation of fold change and dispersion for RNA-seq data with DESeq2. Genome Biol *15*, 550.

Nichterwitz, S., Chen, G., Aguila Benitez, J., Yilmaz, M., Storvall, H., Cao, M., Sandberg, R., Deng, Q., and Hedlund, E. (2016). Laser capture microscopy coupled with Smart-seq2 for precise spatial transcriptomic profiling. Nat Commun *7*, 12139.
