# Supplementary material for: Modeling Motor Neuron Resilience in ALS Using Stem Cells
Source: Stem Cell Reports. 2019 May 9;12(6):1329–41. doi: 10.1016/j.stemcr.2019.04.009 (PMC6565614; doi:10.1016/j.stemcr.2019.04.009)
Supplement: Document S2. Article plus Supplemental Information [file mmc6.pdf]

Stem Cell Reports
Article

ISSCR
OPEN ACCESS

Modeling Motor Neuron Resilience in ALS Using Stem Cells

Ilary Allodi,[1,3] Jik Nijssen,[1] Julio Aguila Benitez,[1] Christoph Schweingruber,[1] Andrea Fuchs,[1] Gillian Bonvicini,[1] Ming Cao,[1] Ole Kiehn,[1,2] and Eva Hedlund[1,*]
[1]Department of Neuroscience, Karolinska Institutet, Stockholm, Sweden
[2]Department of Neuroscience, University of Copenhagen, Copenhagen, Denmark
[3]Present address: Department of Neuroscience, University of Copenhagen, Copenhagen, Denmark
*Correspondence: eva.hedlund@ki.se
https://doi.org/10.1016/j.stemcr.2019.04.009

SUMMARY

Oculomotor neurons, which regulate eye movement, are resilient to degeneration in the lethal motor neuron disease amyotrophic lateral sclerosis (ALS). It would be highly advantageous if motor neuron resilience could be modeled *in vitro*. Toward this goal, we generated a high proportion of oculomotor neurons from mouse embryonic stem cells through temporal overexpression of PHOX2A in neuronal progenitors. We demonstrate, using electrophysiology, immunocytochemistry, and RNA sequencing, that *in vitro*-generated neurons are bona fide oculomotor neurons based on their cellular properties and similarity to their *in vivo* counterpart in rodent and man. We also show that *in vitro*-generated oculomotor neurons display a robust activation of survival-promoting Akt signaling and are more resilient to the ALS-like toxicity of kainic acid than spinal motor neurons. Thus, we can generate bona fide oculomotor neurons *in vitro* that display a resilience similar to that seen *in vivo*.

INTRODUCTION

Amyotrophic lateral sclerosis (ALS) is characterized by a loss of motor neurons in cortex, brain stem, and spinal cord with subsequent spasticity, muscle atrophy, and paralysis. Motor neurons innervating the extraocular muscles, including the oculomotor, trochlear, and abducens nuclei, are however relatively resistant to degeneration in ALS (Comley et al., 2016; Gizzi et al., 1992; Nijssen et al., 2017; Nimchinsky et al., 2000). This resilience has been attributed to cell-intrinsic properties (Allodi et al., 2016; Brockington et al., 2013; Comley et al., 2015; Hedlund et al., 2010; Kaplan et al., 2014). It would be highly advantageous if differential motor neuron vulnerability could be modeled *in vitro*. This could aid in the identification of gene targets responsible for oculomotor neuron resilience. However, it is not self-evident that *in vitro*-generated oculomotor neurons would be more resistant to degeneration in culture than spinal motor neurons, as multiple cell types contribute to motor neuron degeneration in ALS, including, for example, astrocytes (Yamanaka et al., 2008), microglia (Boillee et al., 2006), and oligodendrocytes (Kang et al., 2013), and may play a role in selective vulnerability.

During development, distinct neuronal populations are defined through diffusible morphogens with restricted temporal and spatial patterns and subsequent activation of distinct intrinsic transcription factor programs. These programs can be mimicked to specify different CNS regions and neuronal populations from stem cells *in vitro*. When mouse embryonic stem cells (mESCs) are exposed to SHH (sonic hedgehog) signaling and retinoic acid, which ventralize and caudalize the CNS during development, spinal motor neurons constitute 15%–30% of the cells in culture, and interneurons the large remainder (Allodi and Hedlund, 2014; Wichterle et al., 2002). Oculomotor neurons constitute only about 1% of the cells in the midbrain; thus, patterning mESC cultures with fibroblast growth factor 8 (FGF8) and SHH to direct cultures toward a midbrain/hindbrain fate (Allodi and Hedlund, 2014; Hedlund et al., 2008) results in cultures with too few oculomotor neurons to allow for any quantitative analysis. However, when combining morphogens with overexpression of the intrinsic determinant *Phox2a* in neural precursors, a large proportion of ISLET1/2^{+} brain stem motor neurons can be generated (Mong et al., 2014). Furthermore, a combination of the transcription factors *Ngn2*, *Isl1*, and *Phox2a* can specify mESCs into cranial motor neurons (Mazzoni et al., 2013). However, the exact identity of motor neurons generated this way has not been previously demonstrated, and it is currently unclear whether these are indeed oculomotor neurons or rather a mix of brain stem motor neurons, including, for example, trigeminal and facial motor neurons.

Toward the goal of developing stem cell-based *in vitro* models of differential vulnerability we generated cultures with a high proportion of oculomotor neurons through temporal overexpression of PHOX2A in mESC-derived neuronal progenitors and compared these with mESC-derived spinal motor neurons. Careful characterization of these distinct motor neuron populations using immunocytochemistry, electrophysiological recordings, and RNA sequencing demonstrated that mESC-derived oculomotor neurons were highly similar to their *in vivo* counterparts in both rodent and man. Finally, we exposed these to ALS-like conditions using kainic acid and demonstrated that oculomotor neurons were more resilient than spinal

This is an open access article under the CC BY license (http://creativecommons.org/licenses/by/4.0/).

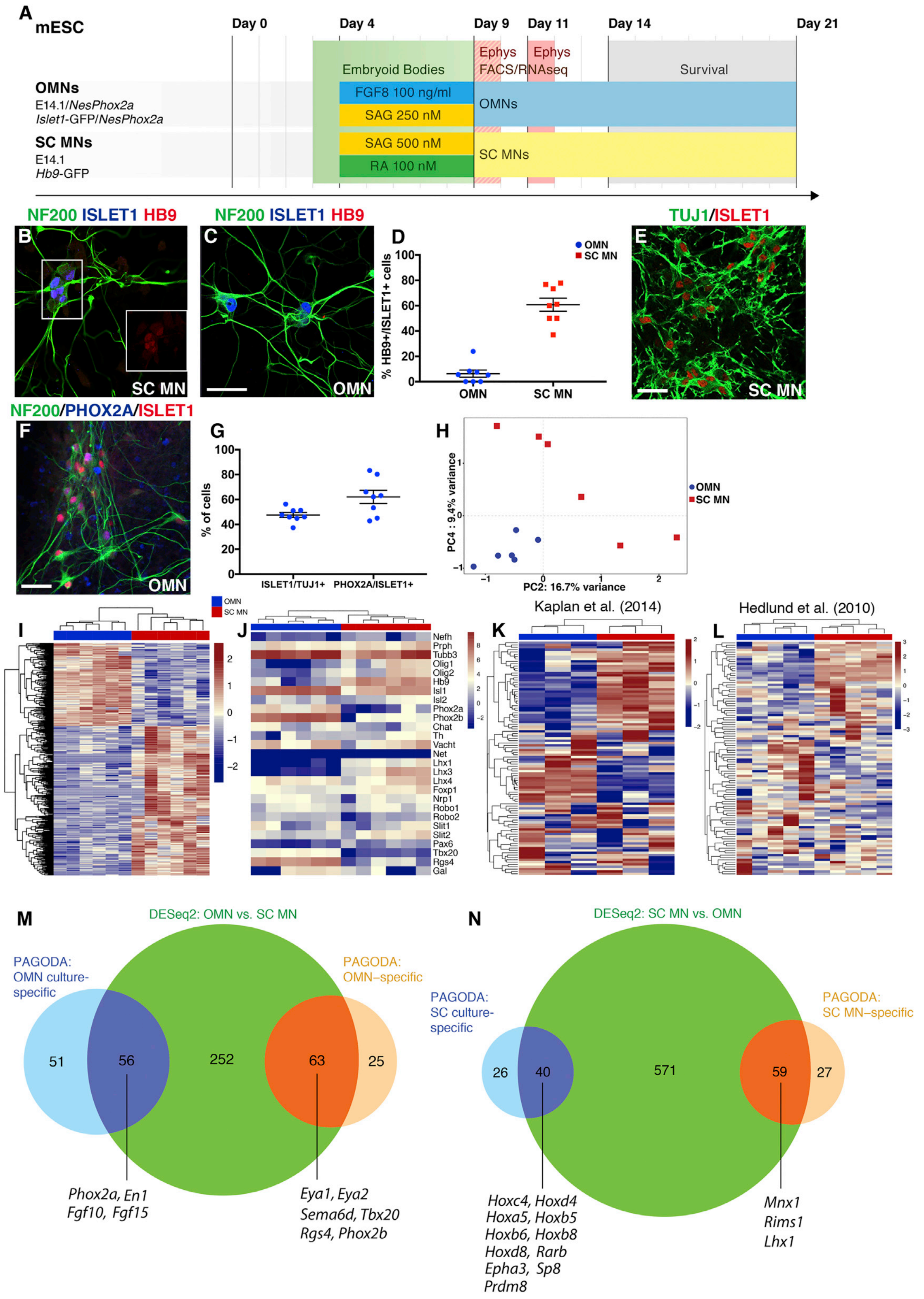

(legend on next page)

motor neurons. Thus, we can generate bona fide oculomotor neurons *in vitro* that display a resilience to ALS similar to that seen *in vivo*.

RESULTS

Bona Fide Oculomotor Neurons Can Be Generated from mESCs

To characterize the specific identity of brain stem motor neurons generated in culture and compare their properties with those of spinal motor neurons, we generated neurons from mESCs. Spinal motor neurons were derived by adding 100 nM retinoic acid and the smoothened agonist SAG to the culture for 5 days. Brain stem motor neurons were specified by overexpressing *Phox2a* under the *nestin* enhancer and patterning the culture with FGF8 and SAG for 5 days (Figure 1A). Immunocytochemical analysis demonstrated that spinal motor neurons expressed the transcription factors ISLET1/2 and HB9 (MNX1) (64.1% ± 5.2% ISLET1/2$^+$HB9$^+$ cells, mean ± SEM, n = 4) (Figures 1B, 1D, and 1E). PHOX2A-overexpressing motor neurons appeared to be of an oculomotor identity as these cells expressed ISLET1/2 but lacked HB9 (6.3% ± 1.3% ISLET1/2$^+$HB9$^+$ cells, mean ± SEM, n = 4), which is present in all cranial motor neuron nuclei except the oculomotor nucleus (Figures 1C, 1D, 1F, and 1G) (Guidato et al., 2003; Lance-Jones et al., 2012). Henceforth we therefore refer to the brain stem motor neurons as oculomotor neurons. In oculomotor neuron cultures 47.5% ± 5.9% (mean ± SEM, n = 4) of βIII-TUBULIN$^+$ cells were ISLET1/2$^+$ and 62% ± 5.2% (mean ± SEM, n = 4) of the ISLET1/2$^+$ cells were PHOX2A$^+$, which demonstrates that 25% of all neurons generated were oculomotor neurons (Figure 1G).

Oculomotor neurons (derived from Islet-GFP mESCs) and spinal motor neurons (derived from Hb9-GFP mESCs) were analyzed for their electrophysiological properties at day-9 and day-11 time points (Figure S1). Cells were able to fire overshooting action potentials in response to depolarizing current pulses. At day 9 few spikes were evoked. Neuronal maturation from days 9 to 11 was seen as a change in the electrical properties whereby day-11 oculomotor neurons could fire trains of action potentials (Figures S1A and S1B) and the width of action potentials was decreased when compared to day 9 (Figure S1C). The increase in the size of neurons was reflected by the increased cell capacity (Figure S1D), decreased input resistance (Figure S1E), and increased rheobase (Figure S1F) from days 9 to 11. Similar changes were seen in spinal motor neurons, which fired a higher number of action potentials (Figures S1G and S1H) and showed a reduced width of action potentials (Figure S1I) at day 11 compared to day 9. Moreover, the changes in cell capacity (Figure S1J), input resistance (Figure S1K), and rheobase (Figure S1L) showed signs of neuronal maturation over time. To further investigate neuronal maturation over time, we performed choline

Figure 1. PHOX2A Overexpression Drives the Generation of Bona Fide Oculomotor Neurons from Stem Cells

(A) Time line depicting *in vitro* protocols for the generation of resistant oculomotor neuron (OMN) and vulnerable spinal cord motor neuron (SC MN). The mESC column reports the four cell lines used in this study. Patterning was performed for 5 days following EB formation. mRNA-seq coupled with FACS was performed on EB dissociation day (day 9 of the protocol). The survival assay was performed 5 days after EB dissociation (day 14 to 21).

(B and C) SC MNs generated from E14.1 ESCs express HB9, ISLET1, and NF200 (B). OMNs generated from E14.1 ESCs overexpressing the transcription factor *Phox2a* under the *Nestin* enhancer co-express ISLET1 and NF200 in the absence of HB9 (C). Scale bar, 60 μm.

(D) Percentage of HB9$^+$/ISLET1$^+$ cells in SC MN (64.1% ± 5.2%, mean ± SEM, n = 4) and OMN (6.3% ± 1.3%, mean ± SEM, n = 4) cultures, respectively (at least 120 Islet1$^+$ cells counted per condition, bar and whiskers represent means ± SEM).

(E) Microphotographs showing ISLET1$^+$/TUJ1$^+$ cells in OMN cultures.

(F) OMNs also express the specific marker PHOX2A as indicated by asterisks. Scale bar, 100 μm (applies also to E).

(G) Quantification of ISLET1$^+$ over TUJ1$^+$ cells demonstrates that half the neuronal population appears to be ISLET1$^+$ (47.5% ± 5.9%, mean ± SEM, n = 4; total number of TUJ1$^+$ cells counted: 1,325). Quantification of PHOX2A$^+$ over ISLET1$^+$ cells (experiments performed in quadruplicates with two technical replicates each, total number of Islet1$^+$ cells counted: 746) indicates that 62% ± 5.2% (mean ± SEM, n = 4) of the ISLET1$^+$ population is also PHOX2A$^+$. All quantifications were performed 5 days after EB dissociation, and experiments were conducted in quadruplicates including two technical replicates per experiment (bar and whiskers represent means ± SEM).

(H) PCA of OMN and SC MN samples based on all genes expressed confirmed cell differential identities. mRNA-seq analysis of OMNs and SC MNs isolated by FACS was performed after 5 days of patterning (day 9 of the protocol).

(I) A total of 1,017 DEGs was found between the two different cell types (adjusted $p < 0.05$, n = 6). Heatmap shows the top 500 most significant DEGs by adjusted p value.

(J–L) Heatmap of expected progenitors and motor neuron transcripts but also OMN and SC MN specific transcripts obtained in the two generated motor neuron populations (J and L). Using the top 100 DEGs obtained from our RNA-seq analysis (K), we could separate datasets originating from *in vivo* microarray studies on early postnatal (J) and adult (L) rodent OMNs and SC MNs.

(M and N) Venn diagrams showing gene sets enriched either in brain stem cultures or OMN-specific as revealed by PAGODA analysis (M), and gene sets preferentially found in spinal cord cultures or MN specific (N).

acetyltransferase (ChAT) staining on oculomotor and spinal motor neuron cultures, confirming expression of the transferase from day 14 *in vitro* in both ISLET1^{+}/HB9^{-} and ISLET^{+}/HB9^{+} cells (Figures S1M and S1N).

To further confirm the identity of the cells generated, we sorted the motor neurons based on GFP expression using fluorescence-activated cell sorting (FACS). For this purpose we used a *Hb9*-GFP mESC line to select for spinal motor neurons and an *Islet*-GFP:*NesEPhox2a* mESCs line for the purification of oculomotor neurons (Figures 1A and S2A–S2D). Poly(A)-based RNA sequencing of the sorted fractions revealed a similar number of detected genes for both lines (reads per kilobase per million mapped reads [RPKM] >0.1: oculomotor neurons 13,941 ± 293.5 and spinal motor neurons 14,660.3 ± 304.1; RPKM >1: oculomotor neurons 10,507 ± 296 and spinal motor neurons 10,914.3 ± 362.7; mean ± SEM, t test for RPKM >0.1 p = 0.1196, t test for RPKM >1 p = 0.4047; Figure S2E).

Sorted oculomotor neurons clustered closely together in the principal component analysis (PCA) and were separated from spinal motor neurons along the fourth principal component (Figure 1H). Hierarchical clustering of the 1,017 genes that were differentially expressed between oculomotor neurons and spinal motor neurons at an adjusted p value of <0.05 demonstrated that each group is defined by a specific set of markers, with a majority of genes being more highly expressed in spinal motor neurons (Figure 1I and Table S1). Analysis of Hox mRNAs clearly demonstrated the more caudal nature of spinal motor neurons compared with oculomotor neurons (Figure S1F). The oculomotor neurons and spinal motor neurons clustered separately based on known marker gene expression, e.g., *Phox2a*, *Phox2b*, *Tbx20*, and *Rgs4* for oculomotor neurons and *Olig1/2*, *Hb9*, and *Lhx1/3* for spinal motor neurons. Oculomotor neuron cultures were also devoid of noradrenergic neuron markers *Slc6a2* (*NET*) and *Th* (Figure 1J).

Using the top 100 differentially expressed genes (DEGs) between our *in vitro*-generated oculomotor neurons and spinal motor neurons, we could separate a published microarray dataset (Mazzoni et al., 2013) of *in vitro*-generated brain stem and spinal motor neurons (Figure S1G). Importantly, we could also use the 100 top DEGs between our oculomotor and spinal motor neurons to separate microarray data originating from mouse oculomotor and spinal motor neurons isolated from postnatal day 7 (P7) mice (Kaplan et al., 2014) (Figure 1K) and from adult rats (Hedlund et al., 2010) (Figure 1L). Thus, our *in vitro*-generated motor neuron populations closely resemble their *in vivo* counterparts.

To further delineate the nature of our *in vitro* culture systems, we set out to identify gene clusters regulated in groups of samples in an unbiased way. Here we analyzed all GFP-positive (Hb9^{+} for spinal and Islet1^{+} for oculomotor) and -negative fractions from both spinal- and midbrain-specified cultures using PAGODA (Fan et al., 2016). This analysis revealed gene sets enriched in the spinal and oculomotor cultures (GFP^{+} and GFP^{-} samples), as well as in the motor neuron groups specifically (GFP^{+}) (Figures 1M and 1N; Table S1). Of the oculomotor-enriched genes found with DESeq2, a subset was enriched in all midbrain-specified cultures (GFP^{+} and GFP^{-} cells), such as *Phox2a* and Engrailed1 (*En1*). *Fgf10*, known to be expressed in the developing midbrain, including the oculomotor nucleus (Hattori et al., 1997), and *Fgf15*, with known enrichment in midbrain and rhombomere 1 (Partanen, 2007), were also enriched in our midbrain-specified cultures (Figure 1M). The genes that were highly enriched specifically in oculomotor neurons (GFP^{+}) included *Eya1*, *Eya2*, *Tbx20*, and *Phox2b* (Figure 1M). Vice versa, of the genes found to be differentially expressed in spinal motor neurons versus oculomotor neurons with DESeq2, a subset was enriched in all samples of the spinal-specified cultures versus the midbrain-specified cultures. This included Hox genes that regulate positional identity, e.g., *Hoxc4*, *Hoxd4*, *Hoxa5*, *Hoxb5*, *Hoxb6*, *Hoxb8*, and *Hoxd8*, as well as spinal V1 interneuron markers *Sp8* and *Prdm8*. Another subset was found to be highly specific for only the spinal motor neurons, including *Mnx1* (Hb9) and *Lhx1* (Figure 1N).

Different axon guidance-related genes are expressed by oculomotor and spinal cord motor neurons during development (Bjorke et al., 2016; Gutekunst and Gross, 2014; Stark et al., 2015; Wang et al., 2011). To further characterize the *in vitro*-generated oculomotor neurons, we analyzed their mRNA expression of morphological markers that are important for axon guidance. We found that oculomotor neurons were enriched in *Plxna4*, *Sema6d*, *Cdh6*, and *Cdh12*, while spinal motor neurons were enriched for *Epha3*, *Ephx4*, *Sema4a*, and *Sema5b* (Figure S2G). We subsequently analyzed the expression level of these axon guidance RNAs in microarray data from mouse oculomotor and spinal motor neurons isolated from P7 mice (Kaplan et al., 2014) and found that *Sema6d* was preferentially maintained in oculomotor postnatally (Figure S2H).

Thus, our *in vitro*-generated oculomotor neurons have characteristics distinct from spinal motor neurons regarding genes that govern axon guidance, and some of these characteristic are maintained *in vivo*.

In conclusion, we have generated a high proportion of bona fide oculomotor neurons *in vitro* that can be utilized to understand their normal function as well as resilience to degeneration in motor neuron diseases.

Stem Cell-Derived Oculomotor Neurons Are Relatively Resistant to Excitotoxicity

To investigate whether *in vitro*-generated oculomotor neurons are relatively resistant to ALS-like toxicity similarly

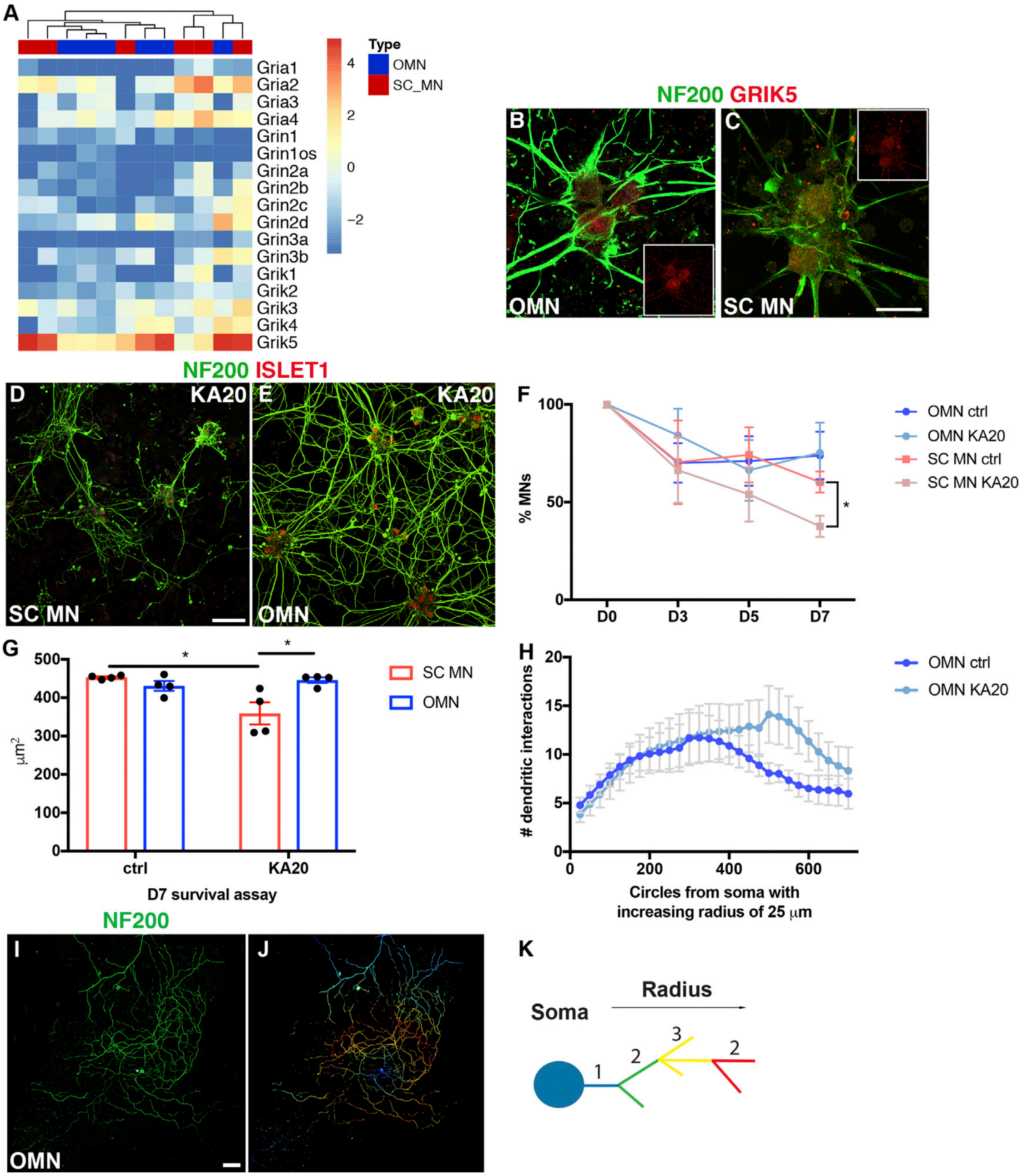

Figure 2. *In Vitro*-Generated Oculomotor Neurons Are Relatively Resistant to ALS-like Toxicity

(A) OMNs and SC MNs express similar mRNA levels of glutamate ionotropic receptor AMPA, NMDA, and kainate type subunits. The heatmap shows $\log_2$ RPKM values of these subunits, and no separate clustering of the two cell types is observed.

(B and C) Immunohistochemistry performed on generated OMNs (B) and SC MNs (C) at D1 *in vitro* in control conditions; similar levels of glutamate ionotropic receptor kainate type subunit 5 (GRIK5) are found in both cell types. Scale bar in (C), 60 μm (applies also to B).

(D and E) Microphotographs presenting SC MN (D) and OMN (E) response to kainic acid-induced toxicity (20 μM) for a week. Scale bar in (D), 100 μm (applies also to E).

(F) Curves represent percentages of MN survival over time in OMN and SC MN cultures. OMNs were visualized as $NF200^{+}ISLET1^{+}HB9^{-}$ cells, while SC MNs as $NF200^{+}ISLET1^{+}HB9^{+}$ cells. OMNs show increased survival to kainic acid toxicity at D7 (bar and whiskers represent means ± SEM, two-way ANOVA and Tukey's multiple comparison test, $F(9, 56) = 2.333$, $^{*}p = 0.0261$, SC MNs $n = 4$, OMNs $n = 5$) when compared with SC MNs (experiments were performed at least in quadruplicate, with technical replicates and with at least 130 motor neurons counted per condition in each experiment).

(legend continued on next page)

to their *in vivo* counterparts, we used kainic acid, which is a neuroexcitatory amino acid that acts by activating glutamate receptors. Glutamate excitotoxicity is thought to be a downstream event in motor neuron degeneration in ALS and is thus considered appropriate to model this disease. First, we evaluated the mRNA levels of glutamate ionotropic receptors in our cultures to ensure that generated neurons could respond to kainic acid. mRNA-sequencing data of sorted motor neurons demonstrated that oculomotor neurons and spinal motor neurons expressed similar levels of AMPA, NMDA, and kainate receptor subunits (Figure 2A). *Grik5* (glutamate receptor, ionotrophic kainate 5) was the subunit expressed at highest level (Figure 2A) and was also detectable at the protein level in both oculomotor (Figure 2B) and spinal (Figure 2C) motor neurons. Thus, both motor neuron types should respond to kainic acid. Exposure of the cultures to kainic acid for 1 week demonstrated that oculomotor neurons were more resilient to the elicited long-term excitotoxicity than spinal motor neurons (ANOVA, $*p < 0.05$; Figures 2D–2F).

Stem cell-derived cultures are not easily temporally restricted in a precise way. Cultures that contain postmitotic neurons may also harbor neuronal progenitors that later on are specified into neurons. It can thus be challenging to distinguish survival from renewal in a stem cell-based *in vitro* system. We therefore analyzed the turnover rate in our cultures by pulsing these with bromodeoxyuridine (BrdU). Analysis of the number of $BrdU^{+}ISLET1/2^{+}$ neurons demonstrated that a significantly higher number of motor neurons were generated over time in the spinal motor neuron cultures compared with the oculomotor neuron cultures (Figure S2I). Consequently, the resilience of oculomotor neurons should be considered even more pronounced as these cultures had a comparatively low turnover rate. Furthermore, we investigated the presence of astrocytes in the cultures and found that only a minority of cells expressed glial fibrillary acidic protein (Figures S2J and S2K). As the processes of motor neurons show the first signs of pathology in ALS, we investigated length of neuronal processes in both oculomotor and spinal motor neuron cultures exposed to kainic acid for 7 days. The result clearly demonstrated that oculomotor neurons were unaffected by kainic acid while spinal motor neurons displayed a shortening of neurites (Figure 2G). To further evaluate oculomotor neurites, we used Sholl analysis to interrogate arborization complexity. The number and branching of oculomotor neurites were not negatively affected under kainic acid treatment (Figures 2H–2K). As axonal fragmentation and degeneration is seen early on in motor neurons in ALS, our data clearly demonstrate that oculomotor neurons cope well under conditions of excitotoxicity and maintain normal morphology.

In conclusion, *in vitro*-generated oculomotor neurons are highly resilient to ALS-like excitotoxicity. Thus, the pattern of selective motor neuron vulnerability seen *in vivo* can be replicated *in vitro*.

Oculomotor Neurons Have High Levels of Calcium-Buffering Proteins and Akt Signaling, which Could Contribute to Resilience

To explain the resilience of *in vitro*-generated oculomotor neurons to excitotoxicity, we conducted a directed comparative analysis of the RNA-sequencing data from purified oculomotor neurons versus spinal motor neurons. It has been suggested that preferential expression of calcium binding proteins in oculomotor neurons plays a role in their resilience (Comley et al., 2015; Van Den Bosch et al., 2002). We focused on transcripts with implications in calcium handling. Our analysis demonstrated that *Cald1*, *Esyt1*, *Camk2a*, and *Hpca11* were preferentially expressed in oculomotor neurons (Figure S3A). ESYT1 protein levels were also preferential to oculomotor neurons and unaffected by kainic acid treatment (Figures S3B–S3D). This could render oculomotor neurons with an increased capacity to buffer Ca^{2+} intracellularly.

We have previously shown that AKT signaling is important in motor neuron resilience toward ALS after insulin-like growth factor-2 treatment (Allodi et al., 2016). We therefore analyzed our RNA-sequencing data from sorted oculomotor and spinal motor neurons for *Akt* signaling effectors, which could aid in explaining the relative resilience of oculomotor neurons *in vitro*. This analysis demonstrated

(G) Analysis of the length of neuronal processes in both oculomotor and spinal motor neuron cultures exposed to kainic acid for 7 days showed that oculomotor neurons were unaffected by kainic acid while spinal motor neurons displayed a shortening of neurites (bar and whiskers represent means ± SEM, two-way ANOVA, $*p < 0.05$, n = 4).
(H–K) Sholl analysis was performed on OMN at D7 survival assay in control and KA20 conditions (H) to further assess individual MN arborization complexity during toxicity. (H) Comparison of the average number of neurite intersections of OMN in control and KA20 toxicity conditions with radial step size of 25 μm. OMNs did not show reduction in arborization (bar and whiskers represent means ± SEM, multiple t test, n = 10). (I) Example of OMN at day 7 of KA20 toxicity. Scale bar in (I), 100 μm (applies also to J). (J) Sholl mask was applied to individual OMNs after specifying the radius from the center of the soma of the neuron and created concentric circles every 25 μm of increasing radius. (K) Schematic depicting identification of neurite segments by Sholl analysis. Color code is assigned depending on arbor localization from the soma in an inside-out manner following the given radius. Multiple intersections within the same segment display the same color.

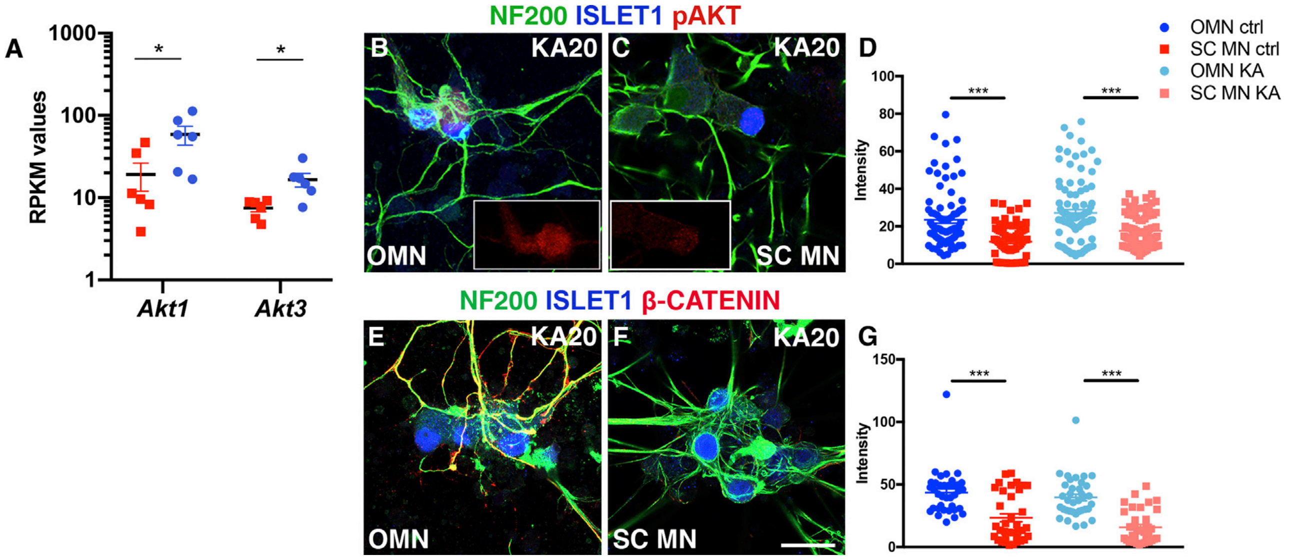

Figure 3. mESC-Derived Oculomotor Neurons Have High Levels of Akt Signaling

(A) RPKM values from RNA sequencing of mESC-derived oculomotor and spinal motor neurons enriched by FACS shows preferential expression of *Akt1* and *Akt3* transcripts in the generated OMNs (bar and whiskers represent means ± SEM, *adjusted $p < 0.05$, $n = 6$).

(B and C) Microphotographs show pAKT levels in OMN (B) and SC MN (C) cultures at D7 toxicity assay assessed by immunohistochemistry, pAKT staining alone in small insets (red channel).

(D and F) Fluorescence intensities (D), analyzed in a semi-quantitative manner, indicate higher expression of pAKT in OMNs (F) (bar and whiskers represent means ± SEM, two-way ANOVA and Tukey's multiple comparison test, $F(1, 353) = 66.1$, ***$p < 0.0001$, $n = 4$). Scale bar in (F), 60 μm (applies also to B and C).

(E and G) β-CATENIN expression is maintained in OMN also during kainate toxicity (E). (G) Semi-quantitative analysis of β-CATENIN over NF200 staining intensities in OMNs and SC MNs in kainate conditions (bar and whiskers represent means ± SEM, t test, $t = 6.799$, $df = 69$, ***$p < 0.0001$, $n = 3$). Scale bar in (E), 60 μm.

an elevated expression of *Akt1* and *Akt3* in oculomotor neurons compared with spinal motor neurons (adjusted $p < 0.05$, Figure 3A). Consistent with our RNA-sequencing findings, immunocytochemistry against pAKT and quantification of fluorescent intensities in ISLET1/2$^+$ oculomotor and spinal motor neurons derived from mESCs demonstrated that oculomotor neurons had higher levels of pAKT protein than spinal motor neurons (Figures 3B–3D). These levels were maintained after excitotoxicity elicited by kainic acid (two-way ANOVA, ***$p < 0.0001$; Figure 3D).

To further validate this pathway in oculomotor neuron resistance, we analyzed the protein levels of activated β-CATENIN (non-phosphorylated), a downstream effector of AKT signaling, using immunocytochemistry (Figures 3E and 3F). The semi-quantitative analysis demonstrated that oculomotor neurons had elevated levels of β-CATENIN compared to spinal cord motor neurons (two-way ANOVA, ***$p < 0.0001$; Figure 3G). β-CATENIN expression was maintained at relatively high levels in oculomotor neurons compared to spinal motor neurons also during kainate toxicity, with expression preferentially localized to processes (Figures 3F and 3G).

Our data thus indicate that AKT signaling, which is a known survival pathway, could contribute to oculomotor neuron resistance in mouse *in vivo* as well as in a dish.

AKT Signaling Is Elevated in Human Postmortem Oculomotor Neurons

To evaluate whether our *in vitro* data recapitulate motor neuron resistance in human, we used the spatial transcriptomics method LCM-seq (laser-capture microdissection coupled with RNA sequencing) (Nichterwitz et al., 2016, 2018) to analyze individually isolated oculomotor neurons (Figures S4A–S4D) and spinal motor neurons (cervical and lumbar spinal cord) (Figures S4E–S4H) from human postmortem tissues. In addition, we isolated motor neurons from the Onuf's nucleus in the sacral spinal cord (Figures S4I–S4L and Table S2), as these are highly resilient to degeneration in ALS and maintained until end stage of disease (Mannen et al., 1977), similar to oculomotor neurons. Analysis of marker gene expression showed that all motor neuron groups expressed high levels of neurofilaments and *VACHT* (*SLC18A3*) (Figure S4M). Motor neurons also expressed *ISL1/2* and *ChAT*, while being almost devoid of contaminating glial markers, e.g., *SLC1A3*, *AQP4*, *CCL3*,

AIF1, *SOX10*, and *MOG* (Figure S4M). Analysis of the *PHOX2A/B* and *HOX* gene code expression clearly clustered oculomotor neurons away from all spinal motor neuron groups (Figure S4N). PCA of all expressed genes separated oculomotor neurons and spinal motor neurons along PC2, while Onuf's nucleus motor neurons separated out on PC3 (Figure 4A). Analysis of the *PI3K-AKT* signaling pathway (Figure S4O) demonstrated that *AKT3* was elevated in human oculomotor neurons (adjusted $p < 0.05$, Figure 4B), similar to mESC-derived oculomotor neurons (Figure 3A). To analyze whether oculomotor and Onuf's motor neurons shared gene expression that was distinct from other spinal motor neurons (cervical and lumbar), we analyzed DEGs of oculomotor versus spinal and Onuf's versus spinal (Table S3). This analysis showed that the majority of DEGs were unique to each cell type, with 1,025 DEGs unique to oculomotor (553 up- and 472 downregulated compared to other spinal motor neurons) and 921 DEGs unique to Onuf's motor neurons (349 up- and 572 downregulated compared to other spinal motor neurons) (Figure 4C and Table S3). High *AKT3* levels were unique to oculomotor neurons. Elevated *PVALB* (parvalbumin) and *GABRA1* levels were also unique to oculomotor neurons (Figure 4C), consistent with previous findings (Brockington et al., 2013; Comley et al., 2015; Hedlund et al., 2010). Oculomotor and Onuf's shared 214 DEGs, of which 58 were upregulated and 142 downregulated compared to other spinal motor neurons, and 14 of which were regulated in the opposite direction in the two nuclei (Figure 4C and Table S3). *MIF* (macrophage migration inhibitory factor), which is neuroprotective in ALS (Shvil et al., 2018), showed shared elevated expression in both oculomotor and Onuf's motor neurons (Figure 4C). Gene ontology (GO)-term analysis of the identified DEGs revealed a number of GO terms that were upregulated in oculomotor neurons versus the other motor neuron groups, including gluconeogenesis, regulation of membrane potential, anterograde transsynaptic signaling, cation transmembrane transport, neurodevelopment, neuron differentiation, and CNS development (Figure 4D and Table S4). Analysis of GO terms enriched in Onuf's motor neurons included synaptic signaling, anterograde, transsynaptic signaling, NAD biosynthetic process, ATP biosynthetic process, regulated exocytosis, regulation of neurotransmitter levels, and synaptic vesicle localization (Figure 4E and Table S4).

In conclusion, our data indicate that *AKT* signaling is elevated also in adult human oculomotor neurons and may in part underlie their resilience to degeneration in ALS. Notably, while oculomotor and Onuf's nucleus motor neurons mainly show distinct gene regulation, we also identified common pathways that may underlie their joint resilience.

DISCUSSION

In ALS, motor neuron subpopulations show differential vulnerability to degeneration. In particular, cranial motor neurons of the oculomotor, trochlear, and abducens nuclei (cranial nerves 3, 4, and 6), which innervate the extraocular muscles around the eyes, are highly resistant throughout disease progression. In our study we demonstrate that a high proportion of bona fide oculomotor neurons can be generated from mESCs through temporal overexpression of PHOX2A, and that these are relatively resilient to ALS-like toxicity.

Oculomotor neurons are generated in the ventral midbrain through the specification by SHH (secreted from the floor plate and the notochord), FGF8, and WNTs (both secreted by and around the isthmic organizer). These morphogens in turn regulate transcription factors that influence differentiation of ventral midbrain neurons, including oculomotor neurons, dopamine neurons, red nucleus neurons, and GABA-positive interneurons (reviewed in Nijssen et al., 2017). Oculomotor neurons are specified by the transcription factors PHOX2A, PHOX2B, and LMX1B (Deng et al., 2011; Pattyn et al., 1997). PHOX2A is required to drive oculomotor neuron fate as demonstrated by the lack of both oculomotor and trochlear nuclei in *Phox2a* knockout mice (Pattyn et al., 1997). PHOX2A is also sufficient to generate a complete oculomotor complex as shown by studies in chick (Hasan et al., 2010; Pattyn et al., 1997). PHOX2B, on the other hand, is sufficient to induce ectopic generation of oculomotor neurons in the spinal cord, but is not required to induce oculomotor neuron specification in the midbrain (Dubreuil et al., 2000; Pattyn et al., 1997). *In vitro* studies have shown that (1) mESCs can be directly programmed into cranial neurons using the proneuronal gene *Ngn2*, in combination with *Islet1* and *Phox2a* (Mazzoni et al., 2013), and (2) overexpression of either PHOX2A or PHOX2B alone in mESC-derived neural progenitors exposed to SHH and FGF8 can promote a midbrain/hindbrain motor neuron fate (Mong et al., 2014). However, while it was demonstrated that cranial motor neurons were generated *in vitro* it was never previously determined whether oculomotor neurons were specifically produced.

Here, we show that PHOX2A overexpression in neural progenitors in combination with patterning using FGF8 and SHH resulted in the generation of 50% ISLET$^+$ neurons of which more than half were PHOX2A$^+$. The transcriptome and proteome of oculomotor neurons is distinct from other motor neurons (reviewed in Nijssen et al., 2017). Notably, oculomotor neurons can be distinguished by their lack of the transcription factor HB9, which defines other somatic motor neurons (Guidato et al.,

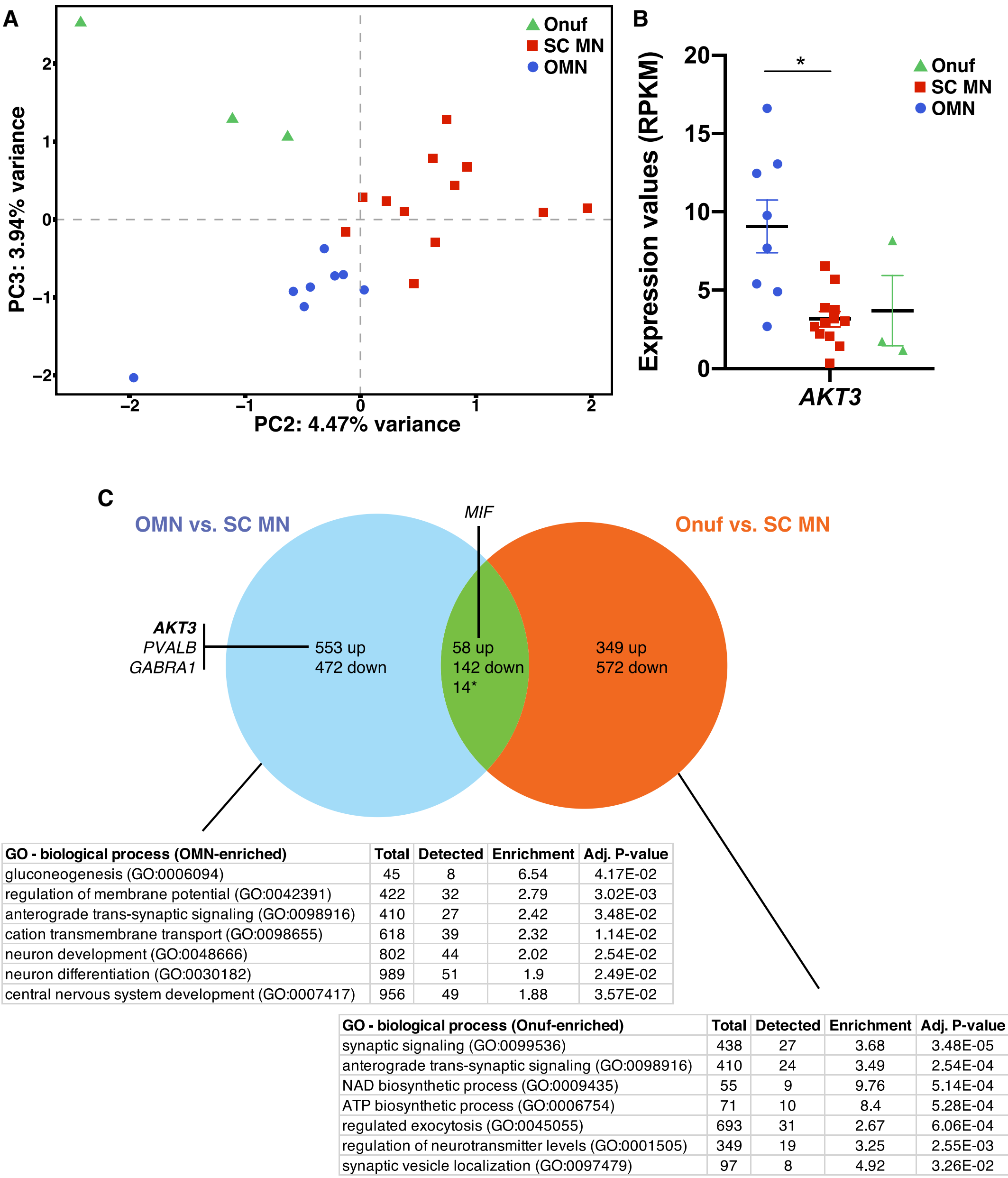

GO - biological process (OMN-enriched)	Total	Detected	Enrichment	Adj. P-value
gluconeogenesis (GO:0006094)	45	8	6.54	4.17E-02
regulation of membrane potential (GO:0042391)	422	32	2.79	3.02E-03
anterograde trans-synaptic signaling (GO:0098916)	410	27	2.42	3.48E-02
cation transmembrane transport (GO:0098655)	618	39	2.32	1.14E-02
neuron development (GO:0048666)	802	44	2.02	2.54E-02
neuron differentiation (GO:0030182)	989	51	1.9	2.49E-02
central nervous system development (GO:0007417)	956	49	1.88	3.57E-02

GO - biological process (Onuf-enriched)	Total	Detected	Enrichment	Adj. P-value
synaptic signaling (GO:0099536)	438	27	3.68	3.48E-05
anterograde trans-synaptic signaling (GO:0098916)	410	24	3.49	2.54E-04
NAD biosynthetic process (GO:0009435)	55	9	9.76	5.14E-04
ATP biosynthetic process (GO:0006754)	71	10	8.4	5.28E-04
regulated exocytosis (GO:0045055)	693	31	2.67	6.06E-04
regulation of neurotransmitter levels (GO:0001505)	349	19	3.25	2.55E-03
synaptic vesicle localization (GO:0097479)	97	8	4.92	3.26E-02

Figure 4. Human Oculomotor Neurons Show a Transcriptional Profile, with Elevated *AKT3*, Unique from Onuf's Nucleus Motor Neurons

(A) PCA of all expressed genes separated into Onuf's, oculomotor (OMN), and spinal (cervical and lumbar) (SC_MN) motor neuron groups.
(B) RNA-sequencing data showed that *AKT3* was elevated in OMNs compared to the other motor neuron groups (*adjusted $p < 0.05$, OMN $n = 8$, SC_MN $n = 12$, Onuf's $n = 3$, bar and whiskers represent means ± SEM).
(C) To distinguish the number of DEGs that were shared or unique to each resistant neuron group compared to vulnerable spinal motor neurons, we analyzed DEGs between OMN and SC_MN and between Onuf's and SC_MN. This analysis showed that the majority of DEGs were unique to each resistant motor neuron group, with OMNs showing 553 upregulated and 472 downregulated unique DEGs and Onuf's motor neurons showing 349 upregulated and 572 downregulated unique DEGs. Oculomotor and Onuf's shared 58 upregulated and 142 downregulated DEGs compared with vulnerable spinal motor neurons. *14 DEGs were regulated in the opposite direction in the two motor nuclei.

2003; Lance-Jones et al., 2012). As the vast majority of ISLET^{+} cells in our midbrain cultures lacked HB9, we conclude that the PHOX2A^{+} cells generated were indeed oculomotor neurons and that *Phox2a* overexpression is sufficient to induce an oculomotor fate from stem cells *in vitro*. This conclusion was further supported by our

RNA-sequencing experiments on purified oculomotor and spinal motor neuron cultures and our bioinformatics cross-comparison with other *in vitro* and *in vivo* datasets from rodent and man (Brockington et al., 2013; Hedlund et al., 2010; Kaplan et al., 2014), which confirmed that the oculomotor neurons we generated were molecularly similar to their *in vivo* counterpart. Furthermore, our analysis of axon guidance molecules demonstrated differential expression of distinct morphological markers in oculomotor and spinal motor neurons. Finally, the electrical properties of the mESC-derived motor neurons matured over time in culture, and cells also expressed the cholinergic marker ChAT. Altogether this demonstrates that we can generate bona fide oculomotor neurons.

Furthermore, we found that mRNAs of *Fgf10* and *Fgf15* were enriched in our oculomotor cultures. These two morphogens are normally expressed in the developing midbrain, and *Fgf10* is even present within oculomotor neurons themselves (Hattori et al., 1997; Partanen, 2007). It is therefore highly conceivable that these factors are involved in further specifying the cultures. We therefore believe that these factors can be used to further improve the *in vitro* differentiation protocol, but this remains to be further investigated.

Multiple studies have demonstrated that differential motor neuron vulnerability appears to be regulated by cell-intrinsic differences in gene expression (Allodi et al., 2016; Brockington et al., 2013; Hedlund et al., 2010; Kaplan et al., 2014). We therefore hypothesized that *in vitro*-generated oculomotor neurons would be more resilient to ALS-like toxicity than spinal motor neurons. Indeed, our experiments on neuronal vulnerability clearly demonstrated that oculomotor neurons were more resistant to the ALS-like toxicity elicited by kainic acid than spinal motor neurons. Furthermore, the fact that oculomotor neuron processes remained intact indicates that these cells were indeed highly resilient, as axonal degeneration is an early sign of pathology in ALS.

It has been shown that oculomotor neurons have a higher calcium-buffering capacity than other motor neuron groups (Vanselow and Keller, 2000), which could render the cells more resilient to excitotoxic insults. Our finding that several transcripts with implications for calcium handling were preferentially expressed in stem cell-derived oculomotor neurons as well as oculomotor neurons in man were thus very compelling and consistent with their resilience. ESYT1, for example, is an ER integral membrane protein that presents a cytosolic synaptotagmin-like domain that can bind organelle membranes and a C2 domain able to bind the plasma membrane. These interactions are involved in exchange of lipids and vesicles but also, in the ER, control of Ca^{2+} homeostasis. Thus, ER-plasma membrane junctions can control Ca^{2+} release from ER in response to extracellular Ca^{2+} levels (Giordano et al., 2013), an event that could have a beneficial effect during excitotoxicity.

Furthermore, we have previously shown that AKT signaling plays an important role in induced resistance in vulnerable motor neurons (Allodi et al., 2016). We now demonstrate that *Akt* signaling is more highly activated in resistant oculomotor neurons *in vitro* as well as *in vivo* in human oculomotor neurons. This observation indicates that oculomotor neurons preferentially activate a cell-intrinsic survival program. Notably, cross-comparison of human oculomotor neurons with resilient human Onuf's nucleus motor neurons showed that elevated *AKT* signaling was unique to oculomotor neurons. When comparing human oculomotor and Onuf's motor neurons with vulnerable spinal motor neurons it became evident that the majority of DEGs were unique to each resilient nucleus. However, there were commonly regulated genes across these two resilient nuclei, including *MIF*. MIF was recently shown to inhibit the toxicity of misfolded SOD1 (Shvil et al., 2018), indicating that these two neuron groups may be better at handling toxic aggregation-prone proteins.

In conclusion, we have demonstrated that bona fide oculomotor neurons can be generated from stem cells *in vitro*. We also show that these neurons are relatively resilient to ALS-like toxicity. This finding enables modeling of neuronal resistance and susceptibility in ALS *in vitro*, which could further elucidate mechanisms that could be highly advantageous to activate *in vivo* to rescue vulnerable motor neurons from degeneration.

EXPERIMENTAL PROCEDURES

Ethics Statement

All work was carried out according to the Code of Ethics of the World Medical Association (Declaration of Helsinki) and with national legislation and institutional guidelines. Animal procedures were approved by the Swedish animal ethics review board (Stockholm Norra Djurförsöksetiska Nämnd). Ethical approval for the use of the human post mortem samples was obtained from the regional ethical review board in Stockholm, Sweden (Regionala Etikprövningsnämnden, Stockholm, EPN). Human CNS samples were obtained from the Netherlands Brain Bank (www.brainbank.nl), the National Disease Research Interchange (www.ndriresource.org), and the NIH Neurobiobank with the written informed consent from the donors or next of kin.

Generation of mESC Lines and Specification into Oculomotor and Spinal Motor Neurons

Three different mESC lines were used in this study: E14.1 (ATCC), *Hb9*-GFP (gift from Dr. S. Thams, Karolinska Institutet), and *Islet1*-GFP (gift from Prof. S. Pfaff, UCSD). To increase oculomotor neuron differentiation efficiency, we generated E14.1/*NesEPhox2a* and *Islet1*-GFP/*NesEPhox2a* mESC lines by Lipofectamine (Invitrogen) transfection. *NesEPhox2a* construct was a gift from Prof. J. Ericson

(Karolinska Institutet). As the nestin enhancer was used for overexpression, transgene overexpression is limited to neuronal progenitors. Specification of mESCs into spinal motor neurons was conducted by exposing embryo bodies (EBs) to the smoothened agonist SAG (500 nM, Calbiochem #364590-63-6) and retinoic acid (100 nM, Calbiochem #302-79-4) for 5 days, while oculomotor neurons were patterned by SAG (250 nM) and FGF8 (100 ng/mL, R&D Systems #423-F8) for 5 days. Medium containing morphogens was replaced daily. EB dissociation and motor neuron culture maintenance were carried out as previously described (Wichterle et al., 2002). Oculomotor neurons and spinal motor neurons were kept in Neurobasal medium + Glutamax (Gibco) with a cocktail of trophic factors (5 ng/mL each of glial cell line-derived neurotrophic factor, brain-derived neurotrophic factor, neurotrophin-3, and ciliary neurotrophic factor). All cultures, including survival experiments, were treated with the mitotic inhibitor 5-fluoro-2′-deoxyuridine (final concentration 2 μM) to reduce cell proliferation.

Fluorescent-Activated Cell Sorting and RNA Sequencing of Stem Cell-Derived Neurons

Islet1-GFP/*NesEPhox2a* and *Hb9*-GFP ESCs were differentiated into oculomotor and spinal motor neurons, respectively. GFP^+ motor neurons were sorted, and positive and negative fractions were collected for mRNA sequencing or plated for subsequent immunocytochemistry analysis. FACS was performed using a 100-μm nozzle, a sheath pressure of 15–25 pounds per square inch and an acquisition rate of 1,000–2,000 events per second (Hedlund et al., 2008). 5×10^3 cells were collected in 5% Triton X-100 (Sigma-Aldrich) in water and then prepared for RNA-seq as previously described (Nichterwitz et al., 2016). Samples were sequenced on Illumina HiSeq2000 and HiSeq2500 platforms with read lengths of 43 and 51 bp, respectively.

Laser-Capture Microdissection and RNA Sequencing of Human Motor Neurons

LCM-seq of motor neurons from human postmortem tissues was performed as previously described (Nichterwitz et al., 2016, 2018).

RNA-Seq Data Processing

Reads from cell-culture samples were mapped to the mouse mm10 reference genome with HISAT2 (Kim et al., 2015) using the publicly available infrastructure (available at usegalaxy.org) on the main Galaxy server (Afgan et al., 2016). Human LCM samples were mapped to hg38/GRCh38 (Gencode version V29) using STAR version 2.5.3 (Dobin et al., 2013). Transcript-level counts and RPKM values were obtained using rpkmforgenes.py (Ramskold et al., 2009). After mapping, the basic exclusion criteria were <500,000 mapped reads and, for LCM samples, lack of both motor neuron marker transcripts ISL1 and ChAT.

ALS-like Toxicity and Motor Neuron Survival

Oculomotor and spinal motor neurons were cultured for 5 days and then exposed to kainic acid. Three concentrations of kainic acid were tested: 20, 50, and 100 μM. A concentration of 20 μM was found sufficient to induce consistent motor neuron death also when motor neurons were cultured on astrocytes. Kainate toxicity was induced for 7 days. Quantifications were performed blind to the motor neuron population and toxicity condition. Pictures were taken with a Zeiss LSM700 confocal microscope (40× magnification and 1.5 digital zoom) using the ZEN 2010S SP1 software and then further analyzed by ImageJ software. At least four experiments per condition were quantified in technical duplicates. Twelve random areas were quantified per each coverslip (at least 130 cells were quantified for each condition). To evaluate proliferation and generation of motor neurons, we exposed cultures to pulses of BrdU (Sigma #B5002) for 24 h. BrdU was dissolved in dimethyl sulfoxide (DMSO; Sigma #276855) and added to the cultures at a final concentration of 10 μM. After 24 h of exposure to BrdU, cells were washed twice with fresh culture medium. Proliferation was assessed at day 0 (D0), day 2 (D2), and day 3 (D3) of the survival protocol and all samples were fixed with 4% paraformaldehyde at day 7 (D7). Quantifications were performed in triplicates with technical duplicates.

Morphological Analysis

Arborization and Sholl analysis were conducted utilizing Fiji software. To compare arborization area of oculomotor neurons and spinal motor neurons, we transformed the microphotographs to a grayscale 8-bit image, and assessed quantification after applying a threshold for signal intensity/background correction. Threshold values were kept constant among images, and microphotographs with higher background noise were excluded. Between 10 and 12 pictures per condition per experiment were quantified. Experiments were performed in quadruplicates. The complexity of oculomotor neuron arborization after kainic acid toxicity was obtained by Sholl analysis (O'Neill et al., 2015) by calculating the average of neurites intersecting defined concentric cycles spaced 25 μm from each other starting from the center of the cell body as a function of distance from soma. Individual oculomotor neurons were analyzed after culturing them at lower density (spinal motor neuron analysis could not be performed due to their poor survival at lower densities). Several microphotographs were taken at 40× magnification with a Zeiss LSM700 confocal microscope, mounted with Adobe Photoshop software, and analyzed with Fiji software. The intersection averages were analyzed by multiple t test.

Statistical Analysis

All statistical analyses were performed with GraphPad software unless otherwise specified. Two-way ANOVA was used when taking into account multiple variables and making multiple comparisons, followed by appropriate post hoc analysis. Unpaired Student's t test was used to compare two groups. Further details on statistical tests used can be found in figure legends as well as information on the total number of motor neurons counted per experiment. All experiments were performed at least in triplicate with technical duplicates. All results are expressed as mean ± SEM unless otherwise specified.

ACCESSION NUMBERS

The original RNA-sequencing data from this study are available at the NCBI Gene Expression Omnibus (http://www.ncbi.nlm.nih.gov/geo/) under accession number GEO: GSE118620 (*in vitro*-generated neuron data) and GEO: GSE93939 (human LCM-seq data).

SUPPLEMENTAL INFORMATION

Supplemental Information can be found online at https://doi.org/10.1016/j.stemcr.2019.04.009.

AUTHOR CONTRIBUTIONS

E.H. conceived the study. E.H. and I.A. supervised the project. E.H., I.A., J.N., J.A.B., C.S., A.F., and O.K. designed experiments. I.A., J.N., J.A.B., C.S., A.F., G.B., and M.C. acquired data. I.A., J.N., J.A.B., C.S., A.F., G.B., M.C., O.K., and E.H. analyzed data. E.H. wrote the manuscript with the help of I.A. and J.N. All authors edited and provided critical input on the manuscript. J.A.B. and C.S. contributed equally.

ACKNOWLEDGMENTS

This work was supported by grants from the Swedish Research Council (2011-2651, 2016-02112); EU Joint Programme for Neurodegenerative Disease (JPND) (529-2014-7500); Ragnar Söderbergs stiftelse (M245/11), Åhlén-stiftelsen (mA9/h11, mA5/h12, mB8/h13, mB8/h14, mA1/h15, mA1/h16), Hjärnfonden (FO2018-0027), Ulla-Carin Lindquists stiftelse för ALS forskning, and Petrus och Augusta Hedlunds Stiftelse (M-2018-0876) to E.H. I.A. was supported by a postdoctoral fellowship from the Swedish Society for Medical Research (SSMF) (2015–2017) for this study and is currently supported by a postdoctoral fellowship from Lundbeck Foundation (2018–2021). J.A.B. is currently supported by a postdoctoral fellowship from SSMF (2017–2019). C.S. was supported by an Early Postdoc Mobility fellowship from the Swiss National Science Foundation (172233). O.K. is supported by Novo Nordisk Foundation Laureate Program. We would like to thank the Netherlands Brain Bank (www.brainbank.nl), the National Disease Research Interchange (www.ndriresource.org), and the NIH Neurobiobank for providing human CNS samples for this study. We also would like to thank Peter Simonsson for his excellent work on the graphical abstract.

Received: August 16, 2018
Revised: April 5, 2019
Accepted: April 8, 2019
Published: May 9, 2019

REFERENCES

Afgan, E., Baker, D., van den Beek, M., Blankenberg, D., Bouvier, D., Cech, M., Chilton, J., Clements, D., Coraor, N., Eberhard, C., et al. (2016). The Galaxy platform for accessible, reproducible and collaborative biomedical analyses: 2016 update. Nucleic Acids Res. *44*, W3–W10.

Allodi, I., Comley, L., Nichterwitz, S., Nizzardo, M., Simone, C., Benitez, J.A., Cao, M., Corti, S., and Hedlund, E. (2016). Differential neuronal vulnerability identifies IGF-2 as a protective factor in ALS. Sci. Rep. *6*, 25960.

Allodi, I., and Hedlund, E. (2014). Directed midbrain and spinal cord neurogenesis from pluripotent stem cells to model development and disease in a dish. Front. Neurosci. *8*, 109.

Bjorke, B., Shoja-Taheri, F., Kim, M., Robinson, G.E., Fontelonga, T., Kim, K.T., Song, M.R., and Mastick, G.S. (2016). Contralateral migration of oculomotor neurons is regulated by Slit/Robo signaling. Neural Dev. *11*, 18.

Boillee, S., Yamanaka, K., Lobsiger, C.S., Copeland, N.G., Jenkins, N.A., Kassiotis, G., Kollias, G., and Cleveland, D.W. (2006). Onset and progression in inherited ALS determined by motor neurons and microglia. Science *312*, 1389–1392.

Brockington, A., Ning, K., Heath, P.R., Wood, E., Kirby, J., Fusi, N., Lawrence, N., Wharton, S.B., Ince, P.G., and Shaw, P.J. (2013). Unravelling the enigma of selective vulnerability in neurodegeneration: motor neurons resistant to degeneration in ALS show distinct gene expression characteristics and decreased susceptibility to excitotoxicity. Acta Neuropathol. *125*, 95–109.

Comley, L., Allodi, I., Nichterwitz, S., Nizzardo, M., Simone, C., Corti, S., and Hedlund, E. (2015). Motor neurons with differential vulnerability to degeneration show distinct protein signatures in health and ALS. Neuroscience *291*, 216–229.

Comley, L.H., Nijssen, J., Frost-Nylen, J., and Hedlund, E. (2016). Cross-disease comparison of amyotrophic lateral sclerosis and spinal muscular atrophy reveals conservation of selective vulnerability but differential neuromuscular junction pathology. J. Comp. Neurol. *524*, 1424–1442.

Deng, Q., Andersson, E., Hedlund, E., Alekseenko, Z., Coppola, E., Panman, L., Millonig, J.H., Brunet, J.F., Ericson, J., and Perlmann, T. (2011). Specific and integrated roles of Lmx1a, Lmx1b and Phox2a in ventral midbrain development. Development *138*, 3399–3408.

Dobin, A., Davis, C.A., Schlesinger, F., Drenkow, J., Zaleski, C., Jha, S., Batut, P., Chaisson, M., and Gingeras, T.R. (2013). STAR: ultrafast universal RNA-seq aligner. Bioinformatics *29*, 15–21.

Dubreuil, V., Hirsch, M.R., Pattyn, A., Brunet, J.F., and Goridis, C. (2000). The Phox2b transcription factor coordinately regulates neuronal cell cycle exit and identity. Development *127*, 5191–5201.

Fan, J., Salathia, N., Liu, R., Kaeser, G.E., Yung, Y.C., Herman, J.L., Kaper, F., Fan, J.B., Zhang, K., Chun, J., et al. (2016). Characterizing transcriptional heterogeneity through pathway and gene set overdispersion analysis. Nat. Methods *13*, 241–244.

Giordano, F., Saheki, Y., Idevall-Hagren, O., Colombo, S.F., Pirruccello, M., Milosevic, I., Gracheva, E.O., Bagriantsev, S.N., Borgese, N., and De Camilli, P. (2013). PI(4,5)P(2)-dependent and Ca(2+)-regulated ER-PM interactions mediated by the extended synaptotagmins. Cell *153*, 1494–1509.

Gizzi, M., DiRocco, A., Sivak, M., and Cohen, B. (1992). Ocular motor function in motor neuron disease. Neurology *42*, 1037–1046.

Guidato, S., Barrett, C., and Guthrie, S. (2003). Patterning of motor neurons by retinoic acid in the chick embryo hindbrain in vitro. Mol. Cell. Neurosci. *23*, 81–95.

Gutekunst, C.A., and Gross, R.E. (2014). Plexin a4 expression in adult rat cranial nerves. J. Chem. Neuroanat. *61-62*, 13–19.

Hasan, K.B., Agarwala, S., and Ragsdale, C.W. (2010). PHOX2A regulation of oculomotor complex nucleogenesis. Development *137*, 1205–1213.

Hattori, Y., Yamasaki, M., Konishi, M., and Itoh, N. (1997). Spatially restricted expression of fibroblast growth factor-10 mRNA in the rat brain. Brain Res. Mol. Brain Res. *47*, 139–146.

Hedlund, E., Karlsson, M., Osborn, T., Ludwig, W., and Isacson, O. (2010). Global gene expression profiling of somatic motor neuron populations with different vulnerability identify molecules and pathways of degeneration and protection. Brain *133*, 2313–2330.

Hedlund, E., Pruszak, J., Lardaro, T., Ludwig, W., Vinuela, A., Kim, K.S., and Isacson, O. (2008). Embryonic stem cell-derived Pitx3-enhanced green fluorescent protein midbrain dopamine neurons survive enrichment by fluorescence-activated cell sorting and function in an animal model of Parkinson's disease. Stem Cells *26*, 1526–1536.

Kang, S.H., Li, Y., Fukaya, M., Lorenzini, I., Cleveland, D.W., Ostrow, L.W., Rothstein, J.D., and Bergles, D.E. (2013). Degeneration and impaired regeneration of gray matter oligodendrocytes in amyotrophic lateral sclerosis. Nat. Neurosci. *16*, 571–579.

Kaplan, A., Spiller, K.J., Towne, C., Kanning, K.C., Choe, G.T., Geber, A., Akay, T., Aebischer, P., and Henderson, C.E. (2014). Neuronal matrix metalloproteinase-9 is a determinant of selective neurodegeneration. Neuron *81*, 333–348.

Kim, D., Langmead, B., and Salzberg, S.L. (2015). HISAT: a fast spliced aligner with low memory requirements. Nat. Methods *12*, 357–360.

Lance-Jones, C., Shah, V., Noden, D.M., and Sours, E. (2012). Intrinsic properties guide proximal abducens and oculomotor nerve outgrowth in avian embryos. Dev. Neurobiol. *72*, 167–185.

Mannen, T., Iwata, M., Toyokura, Y., and Nagashima, K. (1977). Preservation of a certain motoneurone group of the sacral cord in amyotrophic lateral sclerosis: its clinical significance. J. Neurol. Neurosurg. Psychiatry *40*, 464–469.

Mazzoni, E.O., Mahony, S., Closser, M., Morrison, C.A., Nedelec, S., Williams, D.J., An, D., Gifford, D.K., and Wichterle, H. (2013). Synergistic binding of transcription factors to cell-specific enhancers programs motor neuron identity. Nat. Neurosci. *16*, 1219–1227.

Mong, J., Panman, L., Alekseenko, Z., Kee, N., Stanton, L.W., Ericson, J., and Perlmann, T. (2014). Transcription factor-induced lineage programming of noradrenaline and motor neurons from embryonic stem cells. Stem Cells *32*, 609–622.

Nichterwitz, S., Benitez, J.A., Hoogstraaten, R., Deng, Q., and Hedlund, E. (2018). LCM-Seq: a method for spatial transcriptomic profiling using laser capture microdissection coupled with PolyA-based RNA sequencing. Methods Mol. Biol. *1649*, 95–110.

Nichterwitz, S., Chen, G., Aguila Benitez, J., Yilmaz, M., Storvall, H., Cao, M., Sandberg, R., Deng, Q., and Hedlund, E. (2016). Laser capture microscopy coupled with Smart-seq2 for precise spatial transcriptomic profiling. Nat. Commun. *7*, 12139.

Nijssen, J., Comley, L.H., and Hedlund, E. (2017). Motor neuron vulnerability and resistance in amyotrophic lateral sclerosis. Acta Neuropathol. *133*, 863–885.

Nimchinsky, E.A., Young, W.G., Yeung, G., Shah, R.A., Gordon, J.W., Bloom, F.E., Morrison, J.H., and Hof, P.R. (2000). Differential vulnerability of oculomotor, facial, and hypoglossal nuclei in G86R superoxide dismutase transgenic mice. J. Comp. Neurol. *416*, 112–125.

O'Neill, K.M., Akum, B.F., Dhawan, S.T., Kwon, M., Langhammer, C.G., and Firestein, B.L. (2015). Assessing effects on dendritic arborization using novel Sholl analyses. Front. Cell. Neurosci. *9*, 285.

Partanen, J. (2007). FGF signalling pathways in development of the midbrain and anterior hindbrain. J. Neurochem. *101*, 1185–1193.

Pattyn, A., Morin, X., Cremer, H., Goridis, C., and Brunet, J.F. (1997). Expression and interactions of the two closely related homeobox genes Phox2a and Phox2b during neurogenesis. Development *124*, 4065–4075.

Ramskold, D., Wang, E.T., Burge, C.B., and Sandberg, R. (2009). An abundance of ubiquitously expressed genes revealed by tissue transcriptome sequence data. PLoS Comput. Biol. *5*, e1000598.

Shvil, N., Banerjee, V., Zoltsman, G., Shani, T., Kahn, J., Abu-Hamad, S., Papo, N., Engel, S., Bernhagen, J., and Israelson, A. (2018). MIF inhibits the formation and toxicity of misfolded SOD1 amyloid aggregates: implications for familial ALS. Cell Death Dis. *9*, 107.

Stark, D.A., Coffey, N.J., Pancoast, H.R., Arnold, L.L., Walker, J.P., Vallee, J., Robitaille, R., Garcia, M.L., and Cornelison, D.D. (2015). Ephrin-A3 promotes and maintains slow muscle fiber identity during postnatal development and reinnervation. J. Cell Biol. *211*, 1077–1091.

Van Den Bosch, L., Schwaller, B., Vleminckx, V., Meijers, B., Stork, S., Ruehlicke, T., Van Houtte, E., Klaassen, H., Celio, M.R., Missiaen, L., et al. (2002). Protective effect of parvalbumin on excitotoxic motor neuron death. Exp. Neurol. *174*, 150–161.

Vanselow, B.K., and Keller, B.U. (2000). Calcium dynamics and buffering in oculomotor neurones from mouse that are particularly resistant during amyotrophic lateral sclerosis (ALS)-related motoneurone disease. J. Physiol. *525*, 433–445.

Wang, L., Klein, R., Zheng, B., and Marquardt, T. (2011). Anatomical coupling of sensory and motor nerve trajectory via axon tracking. Neuron *71*, 263–277.

Wichterle, H., Lieberam, I., Porter, J.A., and Jessell, T.M. (2002). Directed differentiation of embryonic stem cells into motor neurons. Cell *110*, 385–397.

Yamanaka, K., Chun, S.J., Boillee, S., Fujimori-Tonou, N., Yamashita, H., Gutmann, D.H., Takahashi, R., Misawa, H., and Cleveland, D.W. (2008). Astrocytes as determinants of disease progression in inherited amyotrophic lateral sclerosis. Nat. Neurosci. *11*, 251–253.

Stem Cell Reports, Volume *12*

Supplemental Information

Modeling Motor Neuron Resilience in ALS Using Stem Cells

Ilary Allodi, Jik Nijssen, Julio Aguila Benitez, Christoph Schweingruber, Andrea Fuchs, Gillian Bonvicini, Ming Cao, Ole Kiehn, and Eva Hedlund

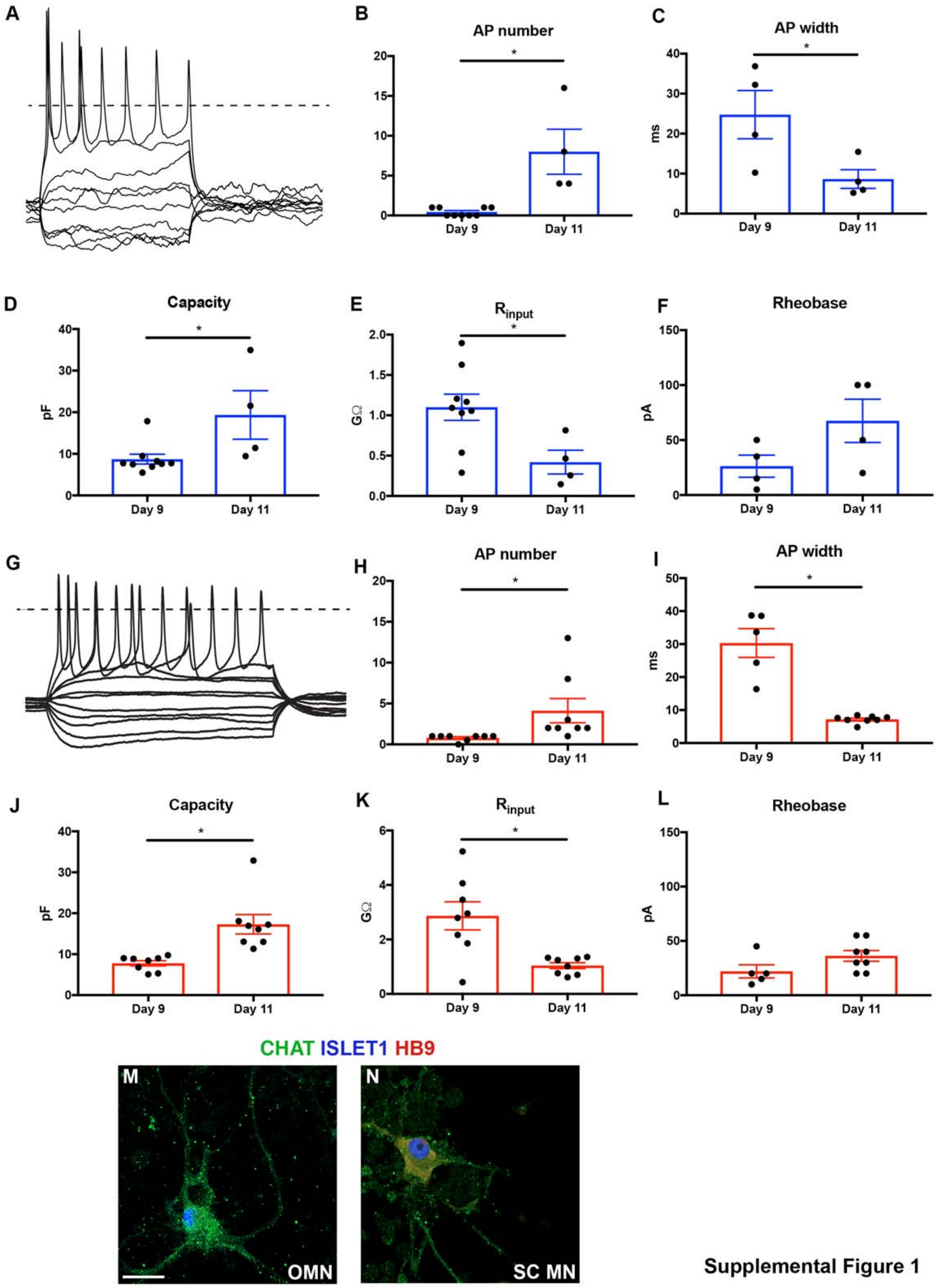

Supplemental Figure 1

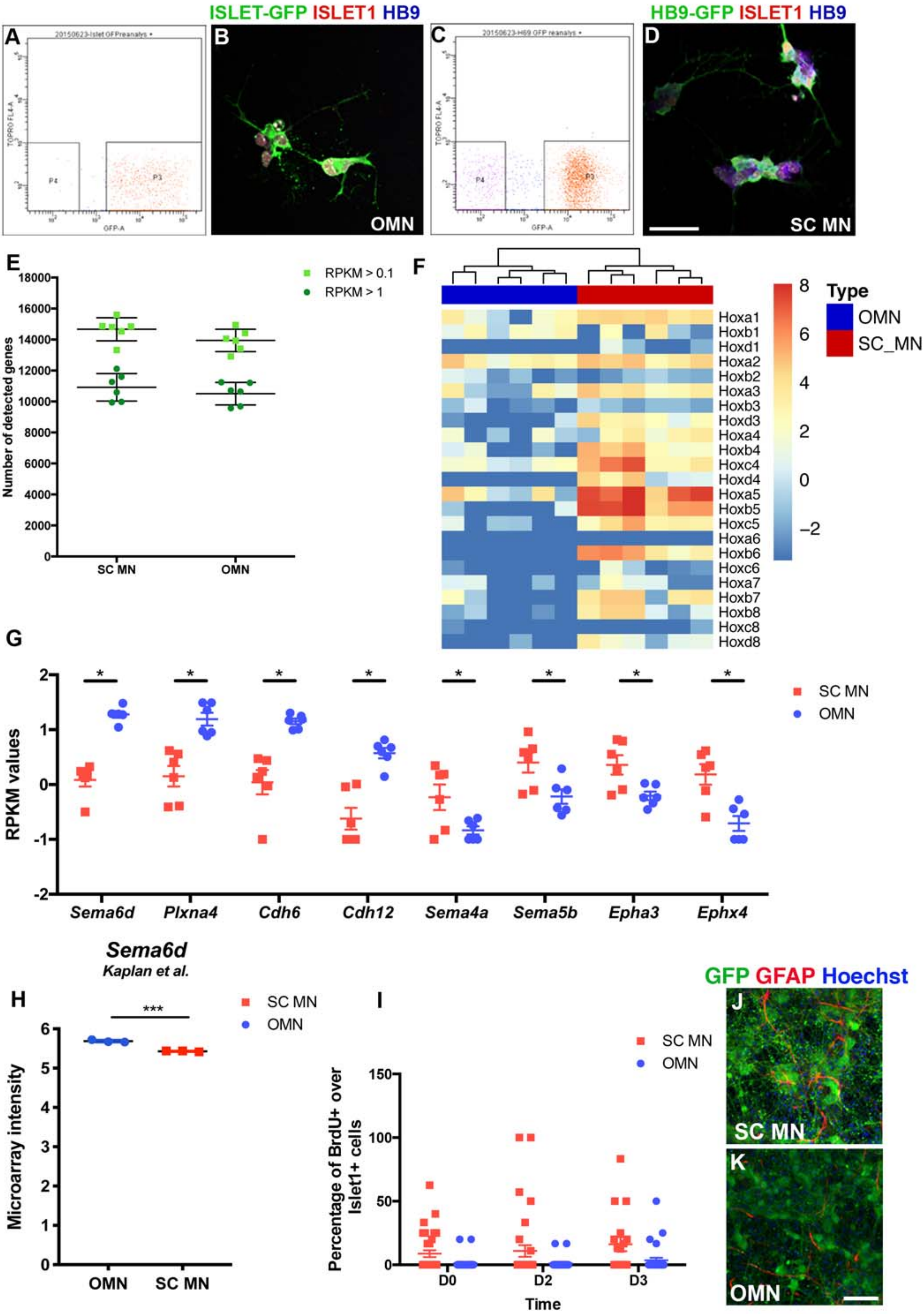

Supplemental Figure 2

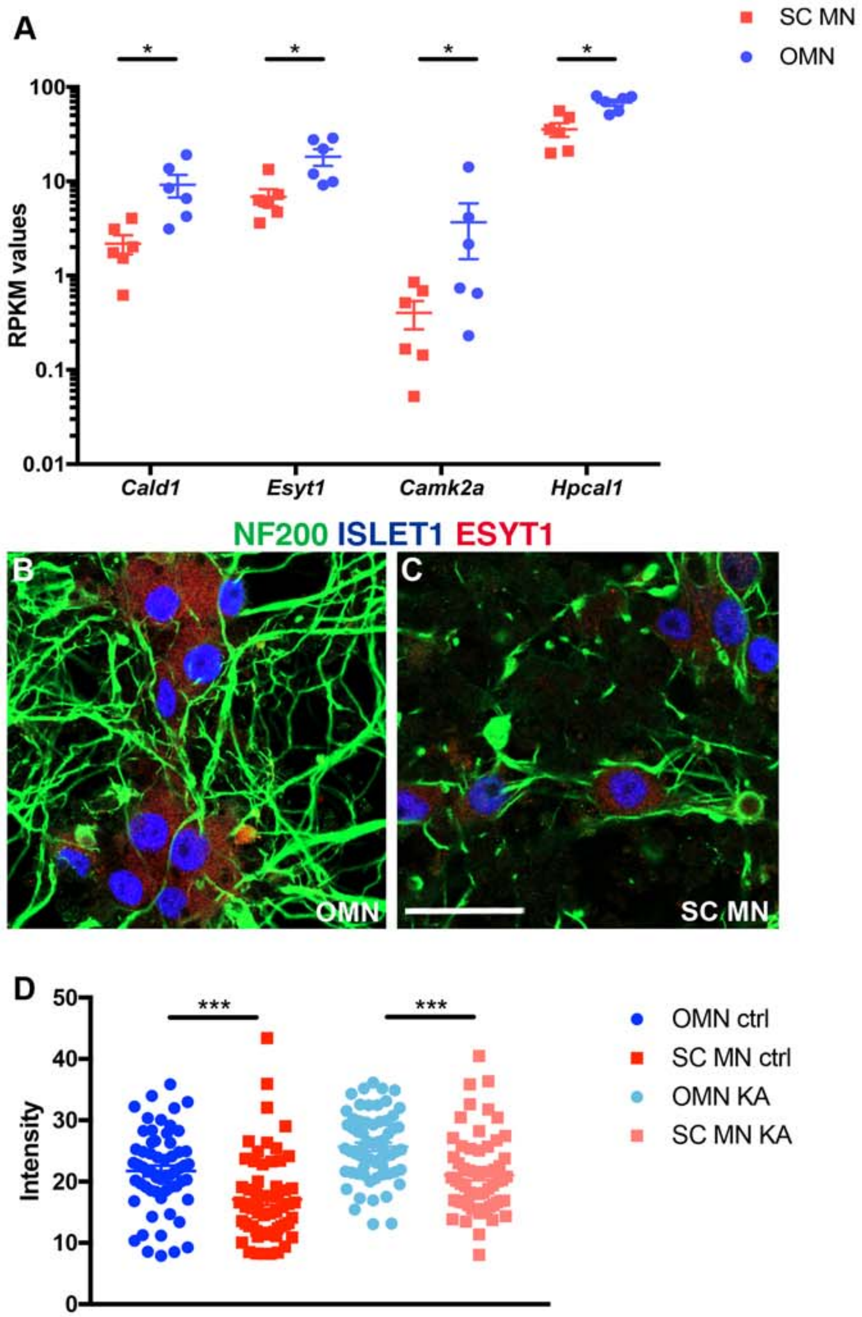

Supplemental Figure 3

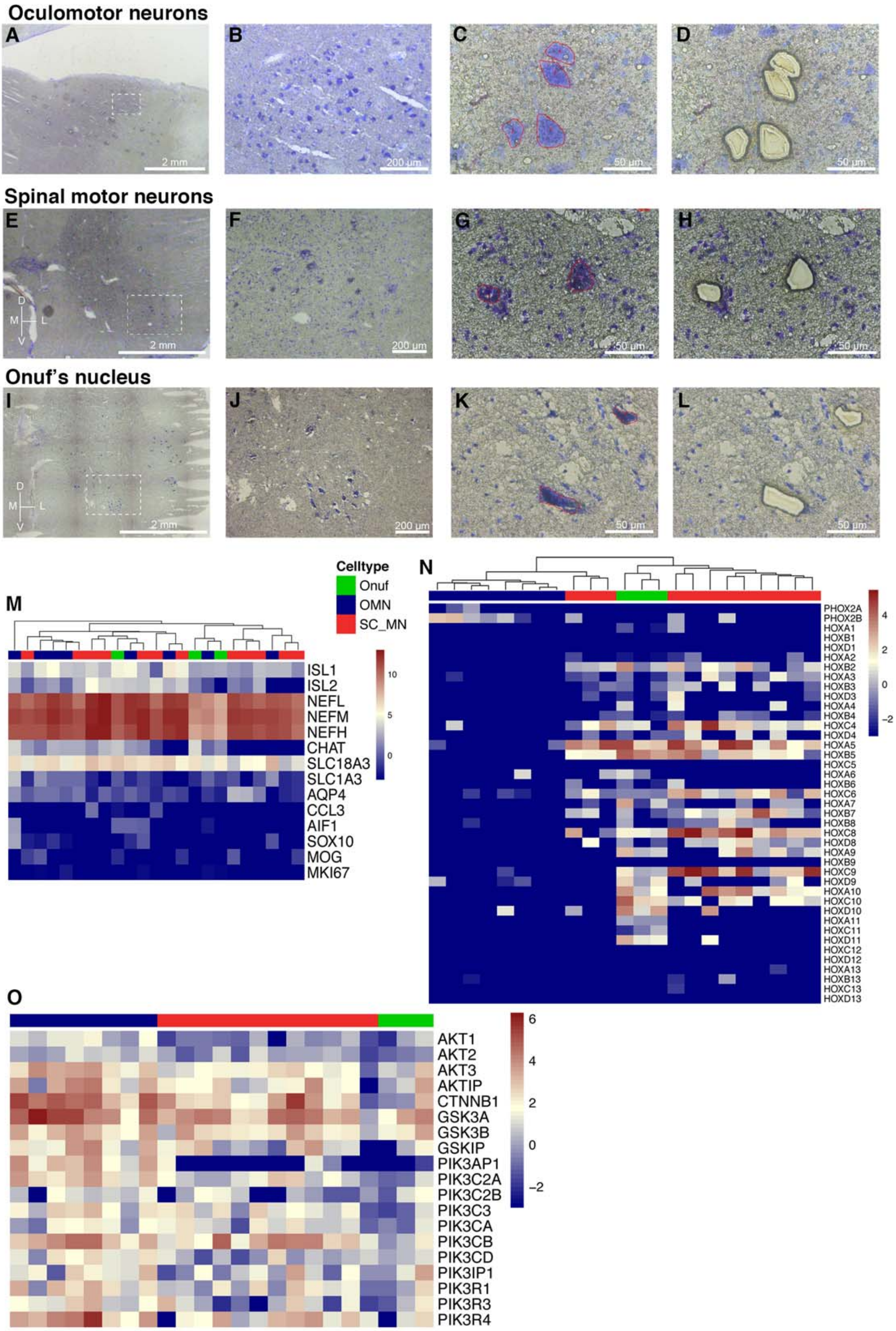

Supplemental Figure S4

Supplemental Figure S1. Oculomotor neurons and spinal motor neurons develop electrical properties and mature in culture. Cells measured in whole cell configuration in current clamp mode, example traces at day 11 for OMN and SC MN cultures (A; G). Dotted lines indicate 0 mV (A; G). Voltage response to step current injections (-40 pA increasing in 10 pA step) at day 11 in OMNs (A). Increased number of action potentials at day 11 compared to day 9 (B, t test, P= 0.0015, mean±SEM) and reduced width of action potentials (C, t test, P=0.0475, mean±SEM). OMNs at day 11 (n=4) show increased cell capacity (D, t test, P=0.0252, mean±SEM), decreased input resistance (E, t test, P= 0.0270, mean±SEM) and increased rheobase (F, t test, mean±SEM) as compared to Day 9 OMNs (n=9). Voltage response to step current injections (-25 pA increasing in 5 pA steps) at day 11 in SC MN (G). Number of action potentials (H, t test, P=0.0428, mean±SEM) and action potential width (I, t test, P<0.0001, mean±SEM) at day 9 and day 11 in SC MNs. Increased cell capacity (J, t test, P=0.0017, mean±SEM), decreased input resistance (K, t test, P=0.0037, mean±SEM) and rheobase (L, t test, mean±SEM) at day 11 as compared to day 9. CHAT staining in OMNs (M) and SC MNs (N) at day 14 in vitro. CHAT in green, HB9 in red, ISLET1 in blue. Scale bar = 60 μm.

Supplemental Figure S2. Generation of oculomotor neurons and spinal motor neurons from stem cells and their characterization. (A) FACS reanalysis plots of *Hb9*-GFP and (C) *Islet1*-GFP/*NesEPhox2*a cells sorted based on GFP expression demonstrated an enrichment of motor neurons (MNs) (*Hb9*-GFP 85%, *Islet*-GFP/*NesEPhox2*a 96% in P3 plots). (B) FAC sorted spinal motor neurons (SC MNs) express ISLET1 and HB9, while oculomotor neurons (OMNs) express ISLET1, while lacking HB9 (D) scale bar = 60 μm. (E) Graph showing mean number of detected genes (and RPKM > 1) (mean ± SEM, t test for RPKM > 0.1 P=0.1196, t test for RPKM > 1 P=0.4047, n=6) after mRNA-seq analysis performed on EB dissociation day following FACS of *Hb9*GFP+ (SC MNs) and *Islet1*GFP/*NesEPhox2*a+ (OMNs) cells. Both conditions show similar amounts of expressed genes. (F) Heatmap showing the differential Hox gene expression between OMNs and SC MNs, confirming their positional identity. (G) DESEQ analysis performed on transcripts obtained from generated oculomotor neurons (OMNs) and spinal motor neurons (SC MNs) reveal significant differences in genes controlling axon guidance, Log_{10}-transformed data for *Sema6d*, *Plxa4*, *Cdh6* and *Cdh12*, preferentially expressed in OMNs, and *Sema4a*, *Sema5b*, *Epha3* and *Ephx4* preferentially expressed in SC MNs (adjusted *P < 0.05, n=6). (H) Preferential expression of *Sema6d* in oculomotor neurons in the published Kaplan et al data set (t test, *P* <0,001). (I) Graph indicated percentage of BrdU+ cells over ISLET1+ cells at D7 assay. BrdU pulses were performed at D0, D2 and D3 survival assay quantifications were performed at D7. (mean ± SEM, 2way ANOVA and Tukey's multiple comparison test, F(2, 169)=0.3278, P=0.7209, n=3). 10 random areas were quantified per each time point per experiment (in duplicates), with at least 100 cells quantified per condition.

Supplemental Figure S3. Preferential expression of Ca^{2+}-regulating genes in oculomotor neurons. (A) After DESEQ analysis of generated oculomotor neuron (OMN) and spinal motor neuron (SC MNs) mRNA expression, several transcripts of Ca^{2+} binding proteins could be found differentially expressed. Graph shows four of them (*Cald1*, *Esyt1*, *Camk2a*, *Hpcal1*) with preferential expression in OMNs suggesting increased capability of intracellular Ca^{2+} buffering during excitotoxicity (adjusted *$P<0.05$, log_{10}-transformed RPKM values, n=6). (B-C) ESYT1 immunohistochemistry performed on OMNs and SC MNs at D7 toxicity assay revealed preferential expression in OMNs at protein level. Scale bar = 60 µm. (D) Semi-quantification of ESYT1 staining in control and KA20 conditions (mean ± SEM, 2way ANOVA and Tukey's multiple comparison test, $F(1, 239)=31{,}84$, *** $P < 0.0001$, n=3). Experiments were performed with technical replicates and with at least 100 motor neurons counted per condition.

Supplemental Figure S4. LCM-seq of human resilient and vulnerable motor neuron groups.

Laser capture microdissection (LCM) was used to isolated oculomotor neurons (A-D), spinal (cervical and lumbar) motor neurons (E-H) and Onuf's nucleus motor neurons (I-L) from human *post mortem* tissues. RNA sequencing of isolated neurons showed that (M) motor neurons also expressed *ISLET1/2* and *CHAT*, while being almost devoid of contaminating glial markers. (N) Analysis of the *PHOX2A/B* and *HOX* gene expression clustered oculomotor neurons away from all spinal motor neuron groups. (O) Analysis of the *PI3K-AKT* signaling pathway.

Supplemental Experimental Methods

Electrophysiology

*Islet1*GFP/*NesEPhox2a* and *Hb9*GFP cell lines were used to visualize oculomotor and spinal cord motor neurons, respectively. EBs were dissociated and cells were plated onto glass coverslips, coated with Poly-L-ornitine (0.001%, Gibco) and fibronectin (1ug/ml, Gibco), at a density of 200,000 cells per well (24-well plate). The coverslips were placed in a recording chamber and where superfused with with oxygenated recording buffer (95% O_2/5% CO_2, 22-24 °C), composed of 111 mM NaCl, 3 mM KCl, 26 mM $NaHCO_3$, 1.1 mM KH_2PO_4, 2.5 mM $CaCl_2$, 1.25 mM $MgCl_2$, and 11 mM D-glucose. Patch pipettes (5-6 MΩ) were pulled from borosilicate glass (GC150F-10, Harvard Apparatus) and filled with (in mM): 128 KGlu, 4 NaCl, 10 HEPES, 0.0001 $CaCl_2$, 1 glucose, 5 ATP, 0.3 GTP, pH 7.4. Neurons were visualized by video microscopy (Axioskop 2 FS plus, Zeiss) using transversal illumination for increasing contrast and motor neurons were identified under fluorescence using a GFP filter. Whole-cell recordings were performed using a MultiClamp 700A amplifier (Molecular Devices) and acquired using pClamp software (Clampex v.9.2, Molecular Devices). Data were sampled at 20 kHz. Input resistance was measured from the I-V curves; the time constant was fitted with a single exponential curve and the capacity was calculated from the cell time constant and input resistance. The width of the action potential was measured at the half peak of the action potential. Rheobase was defined as the minimal current injection needed to elicit an action potential.

Extended laser capture microdissection and RNA sequencing of human motor neurons

Midbrain and spinal cord tissues were sectioned at 10 µm thickness, collected onto MembraneSlide 1.0 PEN microscope slides (Zeiss) and subjected to a quick histological Nissl stain based on the Arcturus Histogene Staining Kit protocol. Slides were then placed under a Leica DM6000R/CTR6500 microscope and captured using the Leica LMD7000 system. Motor neurons were identified by their soma size and their distinct location in axial tissue sections. The cutting outlines were drawn closely around individual cells order to diminish any contamination by surrounding cell. Pools of 20-150 cells were collected and directly lysed in 5µl of 0.2% triton X-100 (Sigma-Aldrich) with RNAse inhibitor (1.5U, Takara). After lysis samples were immediately frozen on dry ice until further processing. Library preparation for sequencing on Illumina HiSeq2000 or HiSeq2500 sequencers was carried out following a modified Smart-seq2 protocol (Nichterwitz et al., 2016).

Extended RNA-seq data processing

For the human LCM data, independent LCM samples obtained from tissue originating from the same individual (biological replicates) were pooled at the counts level. This ensured that each sample used in analysis was derived from one distinct individual. Ultimately, all 12 spinal cord samples were derived

from distinct individuals. Six oculomotor neuron samples originated from distinct individuals, while four more were summed from a total of 12 LCM-seq samples, resulting in a total n=10 samples (from ten individuals) for oculomotor neurons. Two samples were subsequently excluded due to their lack of motor neuron markers, as described above, resulting in a total of eight oculomotor samples. For Onuf's nucleus, three samples (from three individuals) were used in the analysis, derived from a total of seven LCM-seq samples.

Differential gene expression analysis was performed with the DESeq2 package in R on the raw read counts (version 1.16.1) (Love et al., 2014). Only genes with counts in at least 2 samples were considered for further analysis. No fold change cutoff was implemented and multiple testing correction was performed using the Benjamini & Hochberg correction with an FDR set to 10%. Adjusted p-values < 0.05 were considered significant. Pathway And Gene set OverDispersion Analysis (PAGODA) was run using the SCDE package in R (Fan et al., 2016). All heatmaps were generated in R with either no clustering or *Euclidean distance* where appropriate.

Immunocytochemistry and immunohistochemistry

Coverslips were fixed in 4% paraformaldehyde in 0.1M phosphate buffer (4% PFA) for 20 minutes, washed with PBS and then blocked with blocking solution composed of 10% normal donkey serum or normal goat serum (both from Thermo Fisher Scientific Inc.) and 0.1% Triton-X-100 in PBS for one hour at room temperature. Coverslips were incubated overnight with primary antibodies in blocking solution at 4˚C and then incubated with Alexa Fluorophore 488, 568 or 647 secondary antibodies (Invitrogen) one hour at room temperature. The following primary antibodies were used: NF200 (1:1000, #AB5539 Millipore), TUJ1 (1:1000, MRB-435P Covance), HB9 (1:10, #81.5C10 DSHB), ISLET1 (1:500, #ab20670 abcam; 1:100, #40.2D6 DSHB), PHOX2A (1:1000, gift of Prof JF Brunet), GFP (1:1000, #ab13970 abcam), phospho-AKT (Ser473) (1:50, #3787 Cell Signaling), activated BETA-CATENIN (1:1000, #05-665 Millipore), alpha-CATENIN (1:1000, #AB153721 AbCam), ESYT1 (1:100, #HPA016858), BrdU (1:10, #G3G4 DSHB). Hoechst-33342 (Invitrogen) was used as a counterstain. Finally, coverslips were mounted on glass slides using Mowiol 4-88 mounting media (Calbiochem). For immunocytochemistry semi-quantifications and BrdU quantifications at least 3 experiments per condition were counted in technical duplicates. Between 10 and 12 random areas were analyzed per coverslip by blind researcher. For validation of ESYT1 antibody, immunohistochemistry was performed on adult mouse tissue at midbrain and spinal cord levels. Briefly, mice were transcardially perfused with PBS and then with 4% PFA. Brains and spinal cords were dissected and post-fixed for 3 and 1 hour respectively in 4% PFA. Tissue was then cryoprotected with 30% sucrose and then cut at 30 μm thickness. Immunohistochemistry for ESYT1 was performed as indicated above.

Use of published datasets

Microarray data from Kaplan et al. (2014) was obtained through GEO accession number GSE52118.

References

Fan, J., Salathia, N., Liu, R., Kaeser, G.E., Yung, Y.C., Herman, J.L., Kaper, F., Fan, J.B., Zhang, K., Chun, J., *et al.* (2016). Characterizing transcriptional heterogeneity through pathway and gene set overdispersion analysis. Nat Methods *13*, 241-244.

Kaplan, A., Spiller, K.J., Towne, C., Kanning, K.C., Choe, G.T., Geber, A., Akay, T., Aebischer, P., and Henderson, C.E. (2014). Neuronal matrix metalloproteinase-9 is a determinant of selective neurodegeneration. Neuron *81*, 333-348.

Love, M.I., Huber, W., and Anders, S. (2014). Moderated estimation of fold change and dispersion for RNA-seq data with DESeq2. Genome Biol *15*, 550.

Nichterwitz, S., Chen, G., Aguila Benitez, J., Yilmaz, M., Storvall, H., Cao, M., Sandberg, R., Deng, Q., and Hedlund, E. (2016). Laser capture microscopy coupled with Smart-seq2 for precise spatial transcriptomic profiling. Nat Commun *7*, 12139.
